# Clusterin facilitates stress-induced lipidation of LC3 and autophagosome biogenesis to enhance cancer cell survival

**DOI:** 10.1038/ncomms6775

**Published:** 2014-12-12

**Authors:** Fan Zhang, Masafumi Kumano, Eliana Beraldi, Ladan Fazli, Caigan Du, Susan Moore, Poul Sorensen, Amina Zoubeidi, Martin E. Gleave

**Affiliations:** 1 The Vancouver Prostate Centre and Department of Urological Sciences, University of British Columbia, Vancouver, British Columbia, Canada V6H 3Z6; 2 Department of Pathology and Laboratory Medicine, University of British Columbia, Vancouver, British Columbia, Canada V6H 3Z6; 3 These authors contributed equally to this work

## Abstract

We define stress-induced adaptive survival pathways linking autophagy with the molecular chaperone clusterin (CLU) that function to promote anticancer treatment resistance. During treatment stress, CLU co-localizes with LC3 via an LIR-binding sequence within autophagosome membranes, functioning to facilitate LC3–Atg3 heterocomplex stability and LC3 lipidation, and thereby enhance autophagosome biogenesis and autophagy activation. Stress-induced autophagy is attenuated with CLU silencing in *CLU*^−/−^ mice and human prostate cancer cells. CLU-enhanced cell survival occurs via autophagy-dependent pathways, and is reduced following autophagy inhibition. Combining CLU inhibition with anticancer treatments attenuates autophagy activation, increases apoptosis and reduces prostate cancer growth. This study defines a novel adaptor protein function for CLU under stress conditions, and highlights how co-targeting CLU and autophagy can amplify proteotoxic stress to delay cancer progression.

## Abstract (web summary)

The induction of autophagy under stress conditions such as chemotherapy is a contributing factor towards resistance to anticancer therapy. Here, Zhang *et al*. identify the molecular chaperone clusterin as an adaptor that facilitates lipidation of LC3 and autophagosome biogenesis.

## Graphical abstract

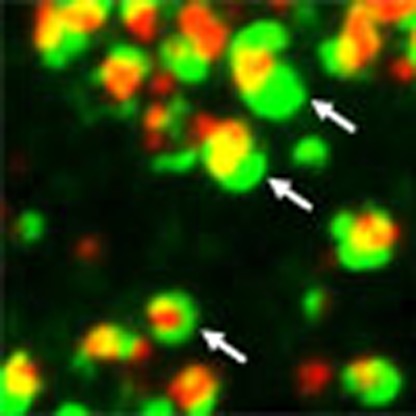

Anticancer treatments induce stress responses that inhibit apoptosis and promote emergence of an acquired treatment-resistant phenotype. Molecular chaperones have key roles in these stress responses, helping maintain protein homeostasis (proteostasis) and regulating signalling and transcriptional survival networks. Chaperones, for instance, help mediate endoplasmic reticular (ER) stress^1^,^2^ and unfolded protein response (UPR) pathways, tailored to re-establish proteostasis by inhibiting translation and promoting proteasome-mediated ER-associated protein degradation (ERAD) and autophagy. While these adaptive responses are cytoprotective, cell death can occur when misfolded protein burden overwhelms the degradation capacity of the proteasome or autophagy^3^,^4^. Co-targeting these stress-induced survival pathways regulating proteostasis may better manipulate cancer cell sensitivity to therapy.

Clusterin (CLU) is a stress-activated chaperone that is transcriptionally regulated by multiple stress-associated factors. CLU expression is stimulated by Egr-1 via IGF-1R–Src–Mek–Erk pathway upon ionizing radiation^5^, and by HSF-1 (ref. 6) and YB-1 (ref. 7) following other cancer treatments. Under stress conditions, CLU retro-translocates from the ER to cytosol^8^ to inhibit apoptosis by suppressing protein aggregation^9^, p53-associated stress signals^10^ and Bax^10^,^11^, while enhancing Akt phosphorylation^12^ and trans-activation of NF-kB^13^, YB-1 (ref. 14) and HSF-1 (ref. 6). CLU is implicated in the pathogenesis of protein aggregopathies and cancer, being highly expressed in Alzheimer’s and treatment-resistant cancer^15^,^16^. In keeping with its anti-apoptotic functions, CLU confers treatment resistance in cancer, while CLU inhibition potentiates activity of anticancer therapies in preclinical models^17^,^18^. The CLU inhibitor, OGX-011 (custirsen, OncoGenex Pharmaceuticals), is in Phase III trials of castrate-resistant prostate cancer (CRPC) and lung cancer after a randomized phase II study in CRPC reported 7-month gain in overall survival and 50% reduced death rate when combined with docetaxel^19^.

Treatment stress can also lead to autophagy activation, an evolutionarily conserved process designed to degrade long-lived proteins and organelles to maintain protein and metabolic homeostasis^20^,^21^. During autophagy, protein aggregates or organelles are sequestered in autophagosomes and degraded in lysosomes to provide recycled building blocks for anabolism and energetics^22^. Although cytoprotective under stress conditions, excessive autophagy can lead to type II programmed cell death^22^. Both CLU and autophagy are associated with protein homeostasis and aggregopathies, tumour suppression in early carcinogenesis, and stress tolerance and treatment resistance in advanced cancer^23^,^24^. Recently, we observed that anticancer treatment induces CLU expression coincident with autophagy activation; however, mechanisms linking stress-induced CLU and autophagy to stress tolerance and treatment resistance are undefined. In this study, we discover that CLU directly interacts with LC3 protein, a key component of autophagy pathway via LC3-interacting region and promotes Atg3–LC3 heterocomplex stability and LC3 lipidation. Furthermore, CLU forms ring-like structures with LC3 on the autophagosome/autolysosome membrane during autophagy biogenesis. Our study defines a novel adaptor protein function of CLU during autophagy activation to support tumour cell survival under cancer treatment stresses.

## Results

### Autophagy-inducing stress stimulates CLU expression

**Figure 1:**
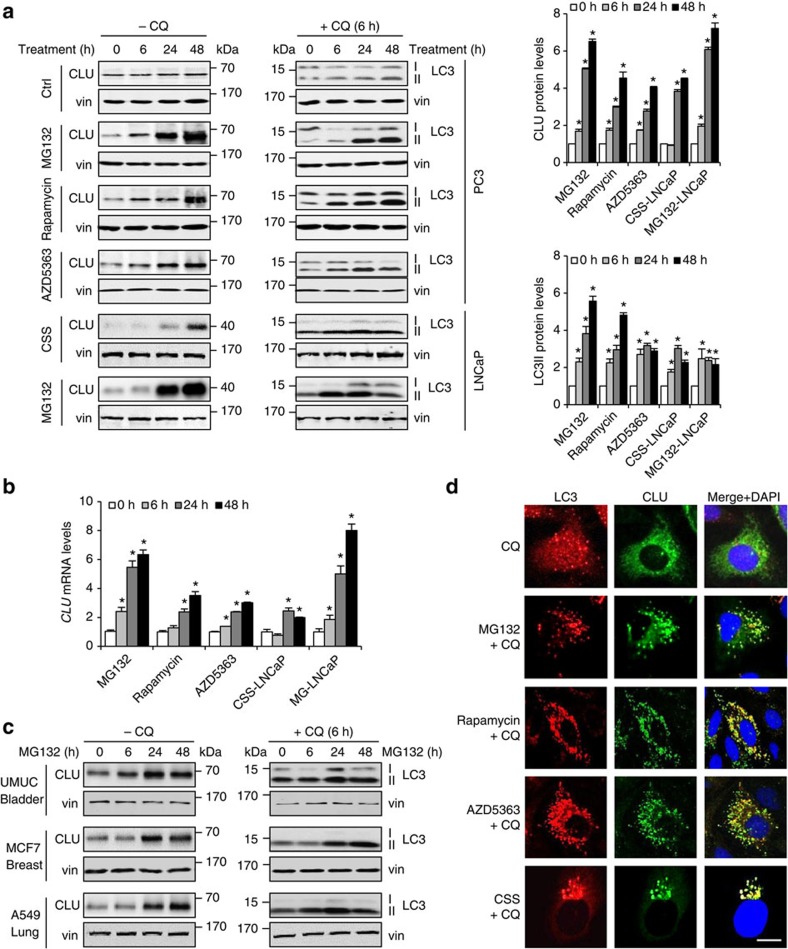
Autophagy-inducing stress triggers CLU expression and co-localization with LC3. (**a**) Autophagy-inducing treatments were applied to PC3 (10 μM of MG132, rapamycin and AZD5363) and LNCaP (CSS and 10 μM MG132) cells ±10 μM chloroquine (CQ) for indicated time periods. Whole protein lysates were collected for western blot against CLU and LC3II; vinculin (vin) was used as loading control. Levels of CLU and LC3II proteins were quantified as shown in the right panels. Statistical analysis were performed compared with the 0 h sample for each treatment group. (**b**) PC3 or LNCaP cells were treated as in **a**, mRNA were extracted and quantitative PCR was carried out to quantify *CLU* mRNA levels. (**c**) Bladder-UMUC, breast-MCF7 and lung-A549 cancer cell lines were treated with 10 μM MG132 ±CQ for indicated time points. CLU and LC3II protein levels were analysed from whole lysates. (**d**) Double immunofluorescence staining for CLU and LC3 was performed in PC3 or LNCaP cells (CSS) treated as in **a** for 6 h. Images were taken using confocal microscope LSM 780. Scale bar, 20 μm. For all panels when indicated, *P<0.05 (Student’s two-tailed *t*-test of three experiments). Error bars: s.e.m of at least three experiments. CSS, charcoal-stripped serum.

To evaluate coordinate changes in CLU expression and autophagy activation after anticancer treatment, levels of CLU and markers of autophagy were initially defined in prostate cancer PC3 cells treated with several known autophagy-inducing agents, including inhibitors of the proteasome (MG132), mTOR (rapamycin) and AKT (AZD5363). Androgen receptor (AR)-positive LNCaP cells were used to assess effects of AR suppression using steroid deprivation with charcoal-stripped serum. Autophagy activity was assessed by western blot for LC3II protein levels in the presence of chloroquine (CQ), which is used to block LC3II degradation in lysosome and, therefore, allow accurate measurement of autophagy flux^25^. In addition to this known effect on autophagy, CQ may also alter proteasome activity. This appearance of phosphatidylethanolamine (PE)-conjugated LC3II protein as puncta or on western blot are established markers of autophagy activity^26^. LC3II protein levels, in parallel with CLU protein (Fig. 1a) and mRNA (Fig. 1b) levels, were induced under all treatment conditions. Induction of CLU in parallel with autophagy activation was also observed in UMUC-urothelial, MCF7-breast and A549-lung cancer cells (Fig. 1c). Autophagy activity was further assessed by visualizing stress-induced LC3 puncta in PC3 cells using confocal microscopy; all treatments not only induced LC3-puncta formation, but also triggered co-localization of CLU with LC3 puncta (Fig. 1d).

### CLU silencing attenuates autophagy activation

**Figure 2:**
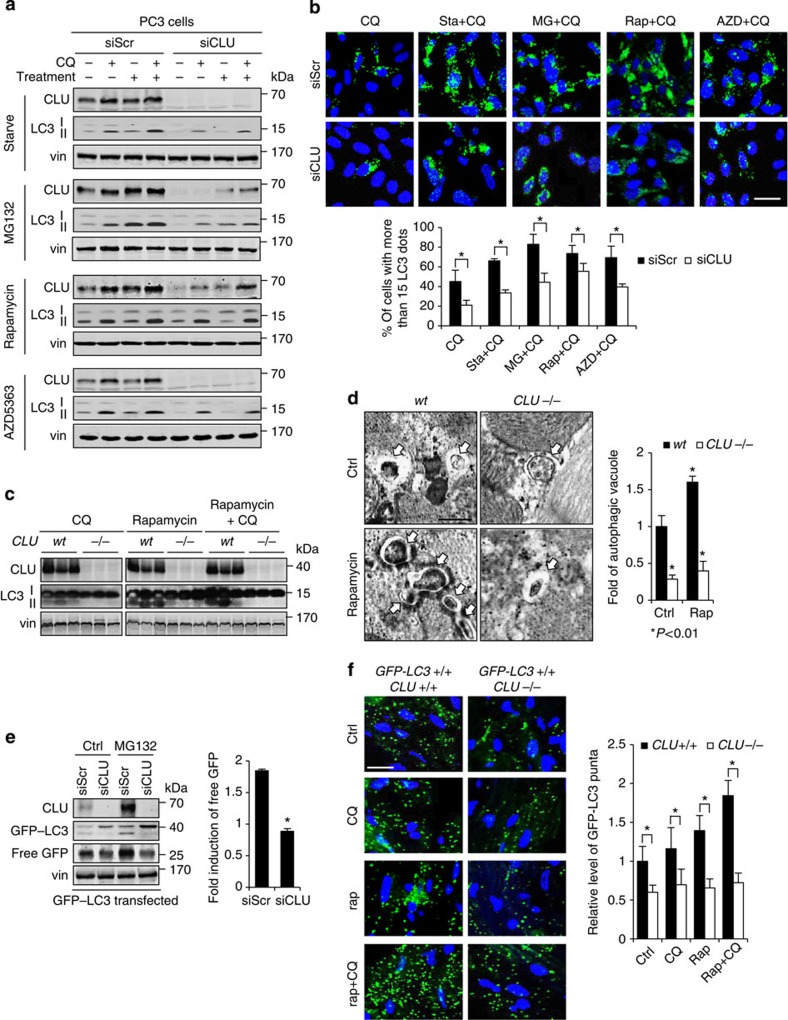
CLU silencing attenuates autophagy activation. (**a**,**b**) PC3 cells were transfected with siCLU or siScr followed by autophagy-inducing stress treatment (nutrient starvation with HBSS/HEPES, 10 μM MG132, rapamycin or AZD5363). Autophagy activity was determined with western blot for LC3II protein (**a**) or microscopy for LC3 puncta (**b**). *P<0.05 (Student’s two-tailed *t*-test of three experiments). Scale bar, 50 μm. (**c**) *CLU* knockout (*−/−)* and wild-type (wt) mice were treated with rapamycin (rap) 10 mg kg^−1^ intraperitoneally ±CQ (10 mg kg^−1^) for 6 h and heart tissues were harvested for western blot for CLU and LC3. (**d**) Heart tissues from mice treated as in **c** were prepared for transmission electron microscope (TEM). Representative images are shown in the left panel, and quantitative data is in the right panel. Statistical analyses were performed compared with control group in wild-type mice. *P<0.01 (Student’s two-tailed *t*-test of three experiments). Scale bar, 500 nm. (**e**) PC3 cells expressing GFP–LC3 were transfected with siCLU or siScr followed by treatment with 10 μM MG132 for 24 h to induce autophagy. The cleavage of GFP from GFP–LC3 was assessed with western blot against GFP protein. The induction of free GFP was examined by comparing the intensity of free GFP protein in MG132-treated versus control samples for siScr or siCLU group. The control sample for both siScr and siCLU were set to 1. *P<0.01 (Student’s two-tailed *t*-test of three experiments). (**f**) *GFP–LC3 x CLU*^*−/−*^ as well as the *wt* (*CLU*^*+/+*^) mice were treated with rapamycin and CQ as in **c**. Mice heart tissues were processed for confocal microscopy for GFP–LC3 puncta. Quantitative data are shown in the right panel. Scale bar, 50 μm. *P<0.01 (Student’s two-tailed *t*-test). Error bars: s.e.m of at least three experiments.

To define functional interactions between stress-induced CLU and autophagy, CLU was silenced in PC3 cells and autophagy activity was evaluated after treatment with nutrient starvation, MG132, rapamycin or AZD5363. CLU siRNA (siCLU) significantly reduced stress-induced LC3II protein levels (Fig. 2a) and LC3-puncta formation (Fig. 2b) under all treatment conditions. Similarly, the antisense CLU inhibitor, OGX-011, blocked treatment-induced CLU and LC3II protein levels (Supplementary Fig. 1a). To assess the effects of CLU silencing on physiologic autophagy induction, *CLU*^−/−^ and wild-type mice were treated with rapamycin plus CQ for 6 h and heart tissues were excised for western blot and transmission electron microscopy (TEM) analysis^27^. Compared with wild-type mice, autophagy activation was dramatically reduced in *CLU*^−/−^ mice, as measured by lower levels of LC3II protein on western blot (Fig. 2c) and number of autophagic vacuoles on TEM (Fig. 2d). In addition, the GFP–LC3 cleavage assay, which measures the degradation of autophagy substrate proteins^28^, also demonstrated significantly reduced generation of free GFP (and hence lower autophagy activity) in PC3 cells after CLU silencing (Fig. 2e). The effect of CLU silencing on autophagy activity was also evaluated *in vivo* in GFP–LC3 mice^27^,^29^,^30^ crossed with *CLU*^−/−^ mice. Treatment with rapamycin plus CQ induced GFP–LC3 puncta in wild-type mice; in contrast, GFP–LC3^+/+^*CLU*^−/−^ mice displayed lower basal level of LC3 puncta, as well as a negligible induction by rapamycin (Fig. 2f). Western blot against GFP protein further confirmed that GFP–LC3II (the lower band on the GFP image) was less induced in *CLU*^−/−^ mice (Supplementary Fig. 1b). Collectively, these cell line and *in vivo* physiologic data indicate that stress-induced autophagy is attenuated when the CLU levels are suppressed.

### CLU overexpression enhances autophagy activity

**Figure 3:**
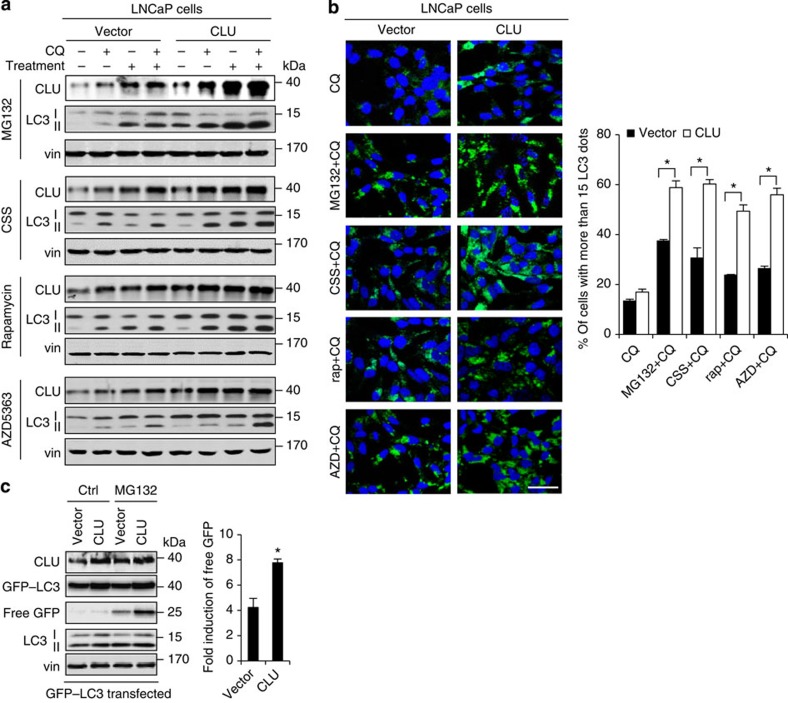
CLU overexpression enhances autophagy activity. (**a**,**b**) LNCaP cells stably overexpressing CLU or vector alone were treated for 24 h with 10 μM MG132, CSS, 10 μM rapamycin or AZD5363 ±CQ. Autophagy activity was assessed using western blot against LC3II (**a**) and microscopy for LC3 puncta (**b**) and quantified in right panel. *P<0.05 (Student’s two-tailed *t*-test of three experiments). Scale bar, 50 μm. (**c**) GFP–LC3 was transfected into CLU-overexpressing or vector-expressing LNCaP cells and treated with 10 μM MG132 for 24 h. The induction of free GFP was examined as Fig. 2e. *P<0.01 (Student’s two-tailed *t*-test of three experiments). Error bars: s.e.m of at least three experiments. CSS, charcoal-stripped serum.

To further assess the role of CLU in autophagy activation, autophagy activity was measured in LNCaP cells (which express low basal levels of endogenous CLU) stably expressing CLU or vector alone. In the presence of CQ, higher induction of LC3II-protein levels (Fig. 3a) and LC3-puncta formation (Fig. 3b) were observed in CLU-overexpressing LNCaP cells. In addition, the GFP–LC3 cleavage assay also showed increased generation of free GFP when CLU is overexpressed (Fig. 3c), suggesting that increased CLU facilitates autophagy activation. We also investigated the role of CLU in mitophagy, a selective autophagy pathway that supports cell survival^31^. CLU was silenced in PC3 cells or overexpressed in LNCaP cells, and then cells were treated with carbonyl cyanide *m*-chlorophenylhydrazone to induce mitophagy. Our data indicate that autophagy activity directly correlates with CLU levels (Supplementary Fig. 2a,b), implicating an additional role for CLU in selective autophagy. These findings are congruent with CLU loss-of-function data in Fig. 2, and support a functional role for CLU in stress-induced autophagy activation.

### CLU forms ring-like structures with LC3 puncta on autophagosome

**Figure 4:**
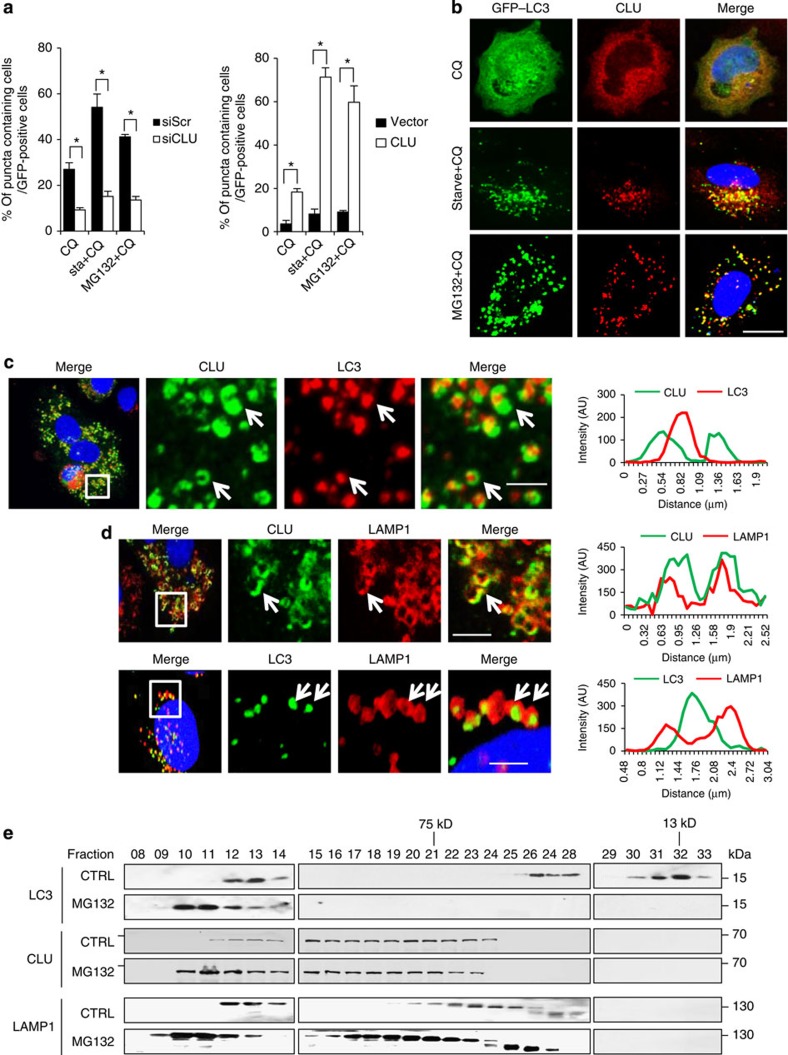
CLU forms ring-like structures with LC3 puncta during autophagosome biogenesis. (**a**) PC3 cells (left panel) treated with siCLU or siScr were transfected with GFP–LC3 plasmid followed by starvation (sta) or MG132+CQ for 6 h. LNCaP cells (right panel) overexpressing CLU or vector were transfected with GFP–LC3 and treated with MG132+CQ for 24 h. Cells were then fixed and GFP–LC3 puncta were analysed and quantified under confocal microscopy. *P<0.05 (Student’s two-tailed *t*-test of three experiments). Error bars: s.e.m of at least three experiments. (**b**) PC3 cells transfected with GFP–LC3 were starved or treated with 10 μM MG132+CQ for 4 h. CLU immunofluorescence staining was performed, and co-localization of CLU and GFP–LC3 were assessed under confocal microscopy. Scale bar, 20 μm. (**c**,**d**) PC3 cells were treated with 10 μM MG132+CQ for 4 h, and double immunofluorescence staining for CLU and LC3, CLU and LAMP1, or LC3 and LAMP1 were carried out. Images were taken with confocal microscopy. Images shown in the right are enlarged from the white boxes in the left images. Profile analysis was performed using ZEN2010 software. Scale bar, 5 μm. (**e**) PC3 cells treated with 10 μM MG132 for 4 h were homogenized and applied for gel filtration assay using a Superdex 200 10/300 GL gel filtration column. Fiver hundred microlitres of fractions were collected and 20 μl aliquots were processed for western blot against CLU, LC3 and LAMP1.

GFP–LC3 protein, which is quenched within the acidic lysosome environment and therefore is only visible in autophagosomes^32^, was used as autophagosome marker to further evaluate the role of CLU in stress-induced autophagosome formation. CLU silencing in PC3 cells reduced, while CLU overexpresson in LNCaP cells enhanced, autophagosome formation as measured by the percentage of GFP–LC3 puncta-containing cells (Fig. 4a and Supplementary Fig. 3a). Furthermore, endogenous CLU in PC3 cells redistributed and co-localized with GFP–LC3 in punctated autophagosomes following treatment stress (Fig. 4b), corroborating findings in Fig. 1d. Interestingly, higher resolution images (Fig. 4c) showed that CLU (in green) forms a ring-like structure surrounding LC3 (in red), suggesting that CLU may localize in the membrane of the autophagosome or autolysosome. To explore this possibility, double immunofluorescence stainings for CLU, LC3 and lysosome-associated membrane protein LAMP1 were performed in MG132- and CQ-treated PC3 cells. High-resolution confocal microscopy clearly illustrated that LAMP1 co-localized with CLU in the ring-like structures (Fig. 4d), confirming CLU localization within autolysosome membranes. In addition, LAMP1 also formed a ring-like structure surrounding LC3 (Fig. 4d), similar to that observed between CLU and LC3 (Fig. 4c). To corroborate these findings, gel filtration analysis on lysates from PC3 cells revealed that CLU co-shifted with LC3 and LAMP1 to large protein complexes (left side of panel) after MG132 treatment (Fig. 4e). In addition, live cell imaging (Supplementary Fig. 3b, Supplementary Movie 1) clearly illustrated dynamic interaction between CLU and LC3 under stress conditions. Collectively, these findings indicate that CLU interacts with LC3 in the regulation of, and integration into, autophagosome membranes.

Next, to assess whether CLU shuttles out of, or stays within, the autophagosome, fluorescence-recovery after bleach assays were performed to characterize the movement of GFP–CLU and GFP–LC3 proteins. Neither CLU nor LC3 recovered after bleaching, indicating that both proteins remain within the autophagosomes (Supplementary Fig. 3c, top panel). Analysis on mobile factors further confirmed similar movements between CLU and LC3 (Supplementary Fig. 3c, lower panel).

To determine the fate of CLU within autophagosomes and whether it is an autophagy substrate, CLU degradation in the lysosome was investigated using a lysosomal protease inhibitor CQ with or without cycloheximide to block *de novo* synthesis of proteins. CLU protein, but not mRNA level, was increased in PC3 cells within 6 h treatment with CQ (Supplementary Fig. 3d), suggesting that CLU is degraded by the lysosome. In contrast, the proteasome inhibitor MG132 increased CLU at both mRNA and protein levels, and this induction was blocked when protein translation was prevented by cycloheximide (Supplementary Fig. 3d), suggesting that MG132 induces CLU at mRNA level and that CLU is not degraded via proteasome pathway under the tested conditions. Collectively, these biochemical and cell imaging data identify key interactions between CLU and LC3 during autophagosome and autolysosome biogenesis, with subsequent degradation of both proteins via the autolysosome.

### CLU regulates Atg3–LC3 heterocomplex stability

**Figure 5:**
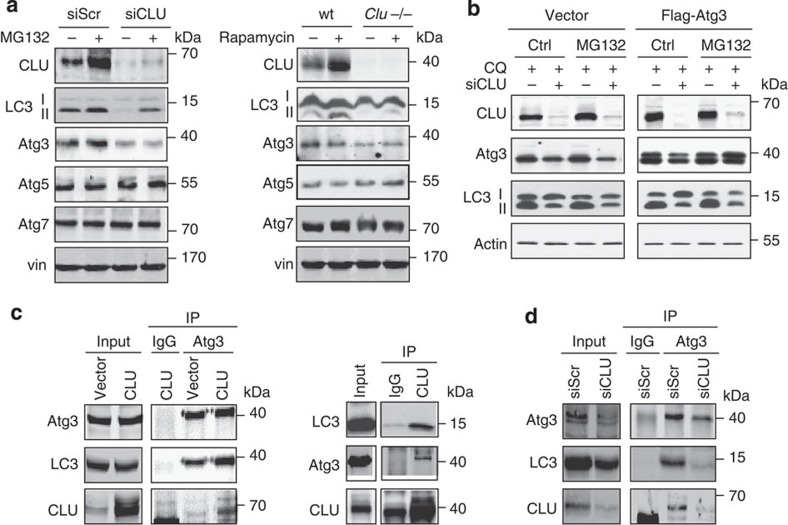
CLU regulates Atg3–LC3 heterocomplex stability and LC3 lipidation. (**a**) PC3 cells transfected with siCLU or siScr were treated with 10 μM MG132 for 6 h. Protein levels were analysed from whole lysates. In the right panel, protein levels were examined from the heart tissue of rapamycin-treated wt or *CLU*^*−/−*^ mice. (**b**) Atg3 plasmid or empty vector was transfected into PC3 cells treated with siCLU, then challenged with 10 μM MG132+CQ for 6 h to induce autophagy. Whole protein lysates were prepared for western blot. (**c**) LNCaP cells were transfected with CLU, Atg3 and LC3 plasmids and then treated with 10 μM MG132+CQ for 6 h. Protein lysates were prepared for co-IP using Atg3 antibody. In the right panel, LNCaP cells were treated as above and co-IP was performed using CLU antibody. (**d**) PC3 cells were transfected with siCLU or siScr, followed by transfection of Atg3 and LC3 plasmids. Cells were treated with 10 μM MG132+CQ for 6 h and protein lysates collected for co-IP using Atg3 antibody.

During autophagy induction, LC3I is conjugated with PE to form LC3II, a key step for autophagosome membrane biogenesis^33^. To define how CLU modulates LC3II conversion and autophagy activity (Figs 2a and 3a), effects of CLU on the expression of Atg family proteins involved in LC3 lipidation was examined. CLU silencing selectively reduced protein level of Atg3, but not other Atg family, in both PC3 cells and heart tissues from *CLU*^*−/−*^ mice (Fig. 5a). Atg3 rescue experiments failed to reverse siCLU-reduced LC3II protein levels, suggesting that lower levels of CLU, rather than Atg3, controlled the reduction of autophagosome formation (Fig. 5b). As Atg3 functions as an E2-like enzyme to facilitate the PE-conjugation to LC3 (ref. 34), and CLU can facilitate SCF-βTrCP E3 ligase activity^13^, we next tested if CLU affects Atg3–LC3 interaction. LNCaP cells were co-transfected with CLU, Atg3 and LC3 plasmids and then treated with MG132+CQ for 4 h. Co-immunoprecipitation (IP) using Atg3 antibody indicated that CLU overexpression increased Atg3–LC3 interaction (Fig. 5c, left panel); moreover, Atg3 also interacted with CLU in co-IP blots (Fig. 5c, right panel), and this was confirmed using reverse IP with CLU antibody (Fig. 5c, right panel). In addition, IP with CLU antibody also revealed interaction of CLU with LC3, consistent with confocal images demonstrating CLU co-localizing with LC3 puncta (Figs 1d and 4c). In contrast, CLU silencing decreased Atg3–LC3 interaction (Fig. 5d). These data suggest that CLU facilitates LC3 lipidation by regulating Atg3–LC3 heterocomplex stability.

### CLU interacts with LC3 through LC3-interacting region

**Figure 6:**
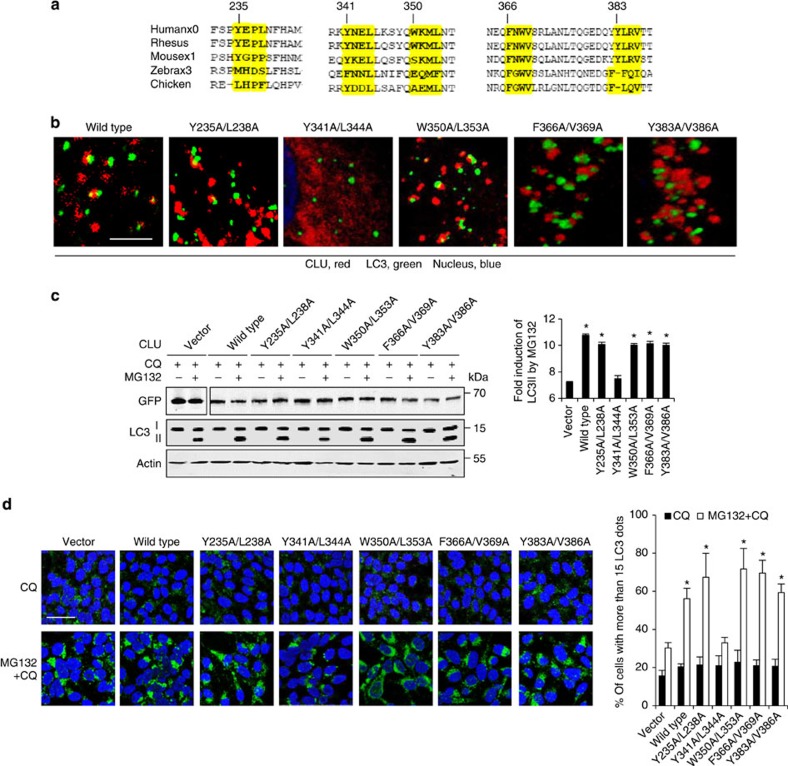
CLU interacts with LC3 via LC3-interacting region to enhance autophagy. (**a**) Sequences of part CLU α-chain protein from various species were aligned to show LIR-like domains. (**b**) CLU wild-type or mutants subcloned into DsRed vector were transfected to PC3 cells. Cells were treated with 10 μM MG132 for 4 h and fluorescence staining on LC3 was carried out. Imaging for DsRed-CLU and green-LC3 were examined using confocal microscopy. Scale bar, 5 μm. (**c**) PC3 cells transfected with wild-type or mutants CLU subcloned to EGFP vector were challenged with 10 μM MG132 for 6 h. GFP–CLU and LC3 protein levels were examined in whole protein lysates. Quantification of LC3II protein was performed and fold induction of LC3II by MG132 compared with ctrl samples were shown (right panel). *P<0.01 (Student’s two-tailed *t*-test of three experiments). (**d**) LNCaP cells transfected with wild-type or mutants CLU were treated with 10 μM MG132±CQ for 24 h and LC3 puncta was analysed. *P<0.05 (Student’s two-tailed *t*-test of three experiments). Scale bar, 50 μm. Error bars: s.e.m of at least three experiments.

LC3-interacting regions (LIR) with the core consensus sequence, W/Y/FxxL/I/V^35^, have been identified in several LC3-interacting proteins such as p62, NDP52, NBR1, Nix, BNIP3 and TP53INP1 (refs 35, 36, 37). We identified five LIR-like sequences in the CLU-α-chain, and alignment analysis indicated high conservation for all five regions (Fig. 6a). Next, wild-type CLU and five LIR mutants were subcloned into DsRed-expressing vector (Supplementary Table 1) and their co-localization with LC3 and LAMP1 were examined in MG132-treated PC3 cells. Among the five mutants, only Y341A/L344A displayed diffuse cytoplasmic imaging that did not co-localize with LC3 puncta (Fig. 6b) or LAMP1 (Supplementary Fig. 4). Expression of this Y341A/L344A mutant failed to enhance LC3II protein levels (Fig. 6c) and LC3-puncta formation (Fig. 6d) post stress compared with wild-type CLU and other LIR mutants. These findings identify the 341YNEL region as a CLU–LIR that mediates CLU–LC3 interaction and facilitates autophagy activation.

### CLU promotes cell survival in part via autophagy pathway

**Figure 7:**
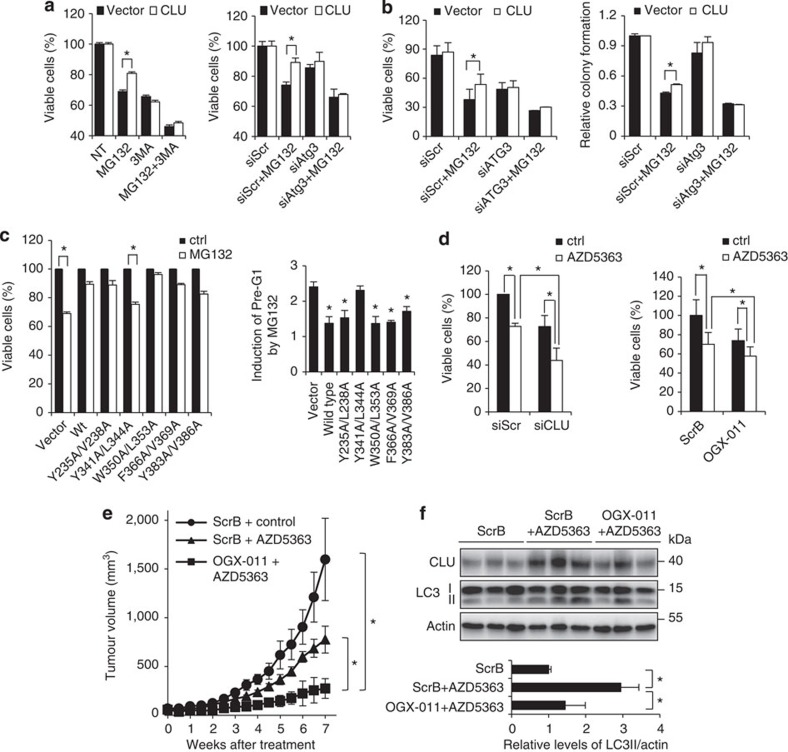
CLU facilitates tumor cell survival via an autophagy-dependent pathway. (**a**) LNCaP cells overexpressing CLU or vector alone were treated with 10 μM MG132±5 mM 3MA for 24 h. Cell viability was determined by crystal violet assay and compared with the non-treated (NT). In the right panel, siRNA targeting Atg3 (siAtg3) instead of 3MA was used to inhibit autophagy activity. (**b**) In the left panel, cells were transfected with siAtg3 and then treated with 1 μM of MG132 and incubated for 5 days before proceedind for crystal violet assay. In the right panel, clonogenic assay were performed with cells transfected with siAtg3 and treated with 1 μM MG132 for 10 days. (**c**) LNCaP cells expressing wild-type or mutants CLU were treated with MG132 for 24 h. Crystal violet assay (left panel) and FACS analysis (right panel) were performed to assess cell death. Fold induction of Pre-G1 by MG132 was calculated by comparing the Pre-G1 percentage from MG132-treated sample to that from non-treated sample for each type of plasmid. (**d**) CLU was silenced in PC3 cells using siCLU or OGX-O11, followed with 20 μM AZD5363 treatment for 72 h. Cell viability was determined as in **a**. (**e**) When PC3 xenografts reached 100 mm^3^, mice were treated with control diluent+ScrB, AZD5363+ScrB or AZD5363+OGX-011 for 7 weeks. Tumour volume were measured twice weekly and calculated by length × width × depth × 0.5236. (**f**) After 1 week of treatment as in **e**, PC3 tumours were harvested for western blot. LC3II levels were quantified and average from the three mice was analysed. For all panels, *P<0.05 (Student’s two-tailed *t*-test of three experiments). Error bars: s.e.m of at least three experiments.

Both autophagy and CLU are induced during stress to degrade toxic protein aggregates and support survival signalling pathways^18^,^38^. To determine whether CLU-promoted cytoprotection relies, in part, on the activation of autophagy, cell viability assays were performed in LNCaP–CLU-overexpressing cells treated with proteotoxic stress (MG132) combined with autophagy inhibition (3-methyladenine, 3MA). While MG132-mediated cell death was partially reduced in LNCaP–CLU-overexpressing cells compared with the vector cells, this cytoprotective effect was attenuated when autophagy was inhibited using 3MA (Fig. 7a, left panel). As 3MA (and other autophagy inhibitors like CQ) may have non-specific actions unrelated to autophagy, siRNA targeting Atg3 was used to specifically inhibit autophagy. Atg3 silencing similarly erased the cell survival gains afforded by CLU under MG132 treatment (Fig. 7a, right panel), indicating that CLU mediates cytoprotection under these conditions in an autophagy-dependent manner. In long-term cell viability assays, CLU overexpression also enhanced survival after MG132 treatment for up to 5 days, and this benefit was attenuated when autophagy was impaired by silencing Atg3 (Fig. 7b, left panel). In addition, clonogenic assays demonstrated more colony formation in LNCaP–CLU-overexpressing cells after MG132 treatment for up to 10 days, and again this effect was blocked when Atg3 was silenced (Fig. 7b, right panel). Concordant with these observations, the CLU Y341A/L344A LIR mutant that failed to trigger autophagy (Fig. 6) also failed to protect cells from MG132-mediated cell death, as shown in cell viability, FACS assays, as well as evaluation of cleaved PARP (Fig. 7c and Supplementary Fig. 5a). CLU inhibition sensitizes autophagy-inducing treatments, as co-targeting CLU (using siCLU or OGX-011) with AZD5363 or MG132 decreased PC3 cell growth and increased apoptotic rates compared with monotherapy (Fig. 7d, Supplementary Fig. 5b). Serum starvation-induced cell death was also increased when CLU was silenced (Supplementary Fig. 5c, left panel), which is similar to the effects of autophagy inhibition using Atg3 siRNA (Supplementary Fig. 5c, right panel).

Previously, we reported that the orally bioavailable Akt inhibitor, AZD5363, potently induces autophagy^39^ and hence is used here to assess *in vivo* effects of CLU inhibition (with OGX-011) on treatment-induced autophagy and tumour growth. While AZD5363-monotherapy delayed PC3 tumour growth compared with controls, combination treatment with OGX-011 significantly delayed tumour growth further (*P*<0.05, Fig. 7e). While AZD5363-monotherapy increased LC3II levels, this effect was attenuated when combined with OGX-011 (Fig. 7f).

Similar effects were also observed when OGX-011 was combined with paclitaxel chemotherapy; repression on CLU expression further delayed tumour growth (Supplementary Fig. 5d) and prolonged survival rate compared with control oligonucleotides (Supplementary Fig. 5e). Evaluation of CLU and LC3II protein levels from these PC3 tumours confirmed that OGX-011 suppressed autophagy activation associated with paclitaxel (Supplementary Fig. 5f). Collectively, these *in vitro* and *in vivo* cell-based studies indicate that co-targeting treatment-induced CLU can suppress stress-induced autophagy activation and delay tumour growth.

## Discussion

Autophagy is a highly conserved degradative pathway that helps cells maintain homeostasis and adapt to proteotoxic, metabolic and other stress^26^,^40^. The relationship between autophagy and cancer is complex and contextual. Early in carcinogenesis, autophagy is tumour-suppressive, reducing accumulation of damaged proteins and genes^38^,^41^. Indeed, defective autophagy can lead to tumour development; for example ATG6/ beclin1, a key gene required for autophagy, is monoallelically deleted in some human breast and ovarian cancers and functions as a haplo-insufficient tumour suppressor in mice^42^. However, autophagy is cytoprotective in established cancer, particularly under stress conditions, facilitating cell survival and adaptation by eliminating toxic protein aggregates and providing sources of nutrients^38^,^41^. Autophagy is induced by physiologic stressors like starvation and hypoxia^39^,^43^ and many anticancer therapies^44^ such as AR pathway inhibition in prostate cancer^45^. In preclinical models, inhibition of autophagy can enhance chemosensitivity and tumour cell death^46^ and clinical studies using the autophagy inhibitor, hydroxychloroquine, are underway^47^,^48^.

Molecular chaperones work in concert with the ubiquitin–proteasome system and autophagy as key regulators of cellular proteostasis mechanisms, assisting misfolded protein refolding or degradation. Consistent with their shared roles in proteostasis, CLU and autophagy are associated with aggregopathies and cancer^49^. CLU is potent inhibitor of protein aggregation^50^ that has an integral part of protein quality-control system regulating recognition and disposal of misfolded proteins. CLU contains amphipathic and coiled-coil helices in addition to large intrinsic disordered regions, properties shared with heat-shock chaperones associated with tissue injury and pathology^51^. CLU is induced by Egr-1, YB-1 and HSF-1 and functions to suppress stress-induced apoptosis^5^,^6^,^7^. Moreover, in cancer, CLU shares with autophagy the paradox of tumour suppressor activity in early-stage carcinogenesis^23^ and promotion of progression of established treatment-resistant cancers^18^,^38^,^41^.

Despite these co-associations, mechanisms linking CLU and autophagy activation to stress tolerance and cancer treatment resistance are undefined. Here, we show that diverse anticancer treatments coincidently induce CLU and autophagy, and CLU silencing in prostate cancer cell lines significantly inhibited stress-induced autophagy and enhanced cell death. Moreover, inhibition of autophagy attenuated CLU-mediated cytoprotection in prostate cancer cell lines, suggesting that CLU supports cancer cell survival partially via autophagy-dependent pathways. Autophagy biogenesis is characterized by the induction, nucleation, extension and completion of an isolation membrane phagophore, regulated by autophagy-related (Atg) genes through ubiquitination-like conjugation steps^29^,^33^. Our studies identify CLU as a key regulator of Atg3-mediated PE-conjugation of LC3I and formation of autophagosome under stress conditions, which is analogous to its role in ubiquitination of COMMD1 and IkB to increase NF-κB activity^13^.

Imaging and biochemical studies demonstrate that upon autophagy activation, CLU co-localizes with LAMP1 in a ring-like structure surrounding LC3. A novel finding in this study is the identification of a functionally relevant LIR in CLU that interacts with LC3. Mutation of this 341YNEL–LIR abrogated stress-induced CLU–LC3 and CLU–LAMP1 co-localization, and LC3 lipidation. Instead of co-localizing with LC3 punta after treatment stress, this CLU–LIR mutant remained dispersed and failed to cytoprotect cancer cells. Specific LIR-containing adaptor proteins, such as p62, function to link ubiquitinated cargoes marked for degradation with autophagosome membranes^31^. Although p62 is the best characterized adaptor protein, others (for example, Nbr1) can also mediate selective autophagy in response to different signals^52^. Indeed, lack of autophagy activation can result from failure to sequester a target protein or adaptor^53^,^54^. Like p62, CLU is associated with autophagy, has a functional LIR site and modifies the activity of other signalling molecules (for example, AKT, NF-κB^55^,^56^).

This study defines a novel adaptor protein function for CLU that enhances autophagy biogenesis under stress conditions by scaffolding LC3I and Atg3 to facilitate PE-conjugation to form LC3II and autophagosomes. Indeed, autophagy induction was dramatically reduced in *CLU*^−/−^ mice, while CLU silencing in prostate cancer cell lines significantly inhibited stress-induced LC3 puncta and enhanced treatment-induced apoptosis. Furthermore, CLU-mediated cytoprotection involves autophagy activation, since the cytoprotective effect of CLU in prostate cancer cells was lost when autophagy was inhibited. CLU has been reported to retrotranslocate from ER during stress to inhibit bax^8^,^11^, but can also promote autophagosome formation to facilitate stress tolerance to anticancer treatment^18^,^49^. Suppression of CLU amplifies proteotoxic stress by reducing treatment-induced autophagy to enhance cell death, illustrating how selective manipulation of stress-induced survival responses can guide biologically rational co-targeting combination strategies.

## Methods

### Cancer cell lines

Human cancer cell lines PC3, MCF7, UM-UC-3 and A549 cells were from the American Type Culture Collection. LNCaP cells were kindly provided by Dr. Leland W. K. Chung (Emery University). LNCaP and UM-UC-3 cells were maintained in RPMI-1640 media (Invitrogen Life Technologies, Burlington, ON) containing 5% heat-inactivated fetal bovine serum (Invitrogen Life Technologies). PC3, MCF7 and A549 cells were maintained in Dulbecco’s modified Eagle’s medium (Invitrogen Life Technologies) containing 5% fetal bovine serum. LNCaP cells stably expressing CLU or empty vector were generated as previously reported^57^. Briefly, LNCaP cells were transfected with CLU-expressing pRC-CMV plasmid or pRC-CMV vector alone, and then selected with 300 μg ml^−1^ of Geneticin (Sigma Chemical Co., St. Louis, MO) for 2 weeks.

### Reagents and antibodies

MG132 was from Calbiochem (Darmstadt, Germany). Chloroquine (CQ), paclitaxel and 3-methyladenine (3MA) were purchased from Sigma-Aldrich. Rapamycin was purchased from LC Laboratories. Akt inhibitor, AZD5363, was kindly provided by AstraZeneca.

Primary antibodies used in this study were: anti-CLU (sc-6419) and anti-LAMP1 (sc-17768) from Santa Cruz Biotechnology; anti-GFP (#2956), anti-cleaved PARP (#5625), anti-Atg3 (#3415), anti-Atg5 (#2630), anti-Atg7 (#2631) and anti-LC3 (LC3B, #2775) from Cell Signaling Technology; and anti-vinculin (V9131) and anti-β-actin (A3853) from Sigma-Aldrich. Secondary antibodies were: anti-goat IgG HRP (sc-2020), anti-mouse IgG HRP (sc-2314) and anti-rabbit IgG HRP (sc-2313) were from Santa Cruz Biotechnology. Secondary antibodies for immunofluorescence staining: donkey anti-rabbit ALexa Fluor 594 (A-21207), donkey anti-goat ALexa Fluor 488 (A-11055) and donkey anti-rabbit ALexa Fluor 488 (A-21206) were all from Invitrogen Life Technologies. Antibodies used for IP were same as above except the one for Atg3 (M133-3) was from Medical &Biological Laboratories (Nagoya, Japan). The dilutions of antibodies are 1:1,000 for primary antibodies and 1:3,000 for secondary antibodies for western blot; and 1:200 for primary antibodies and 1:500 for secondary antibodies for immunofluorescence staining.

### Quantitative reverse transcription PCR

RNA extraction and reverse transcription PCR (RT–PCR) were performed as described previously^6^. Real-time monitoring of PCR amplification of cDNA was carried out using the following primer pairs and probes: *CLU* (Hs00156548_m1) and glyceraldehyde-3-phosphate dehydrogenase (*GAPDH*) (Hs03929097_g1; Applied Biosystems) on the ABI PRISM 7900 HT Sequence Detection System (Applied Biosystems) with TaqMan Gene Expression Master Mix (Applied Biosystems). Target gene expression was normalized to *GAPDH* levels in respective samples as an internal control. The results are representative of at least three independent experiments.

### Transfection

For transfection of plasmids, X-treme GENE9 DNA transfection reagent (Roche, Mannheim, Germany) and Lipofectin 2000 (Invitrogen Life Technologies) was applied for PC3 and LNCaP cells, respectively, according to manufacturer’s user guides. For transfection of siRNA or antisense OGX-O11, oligofectamine (Invitrogen Life Technologies) was used according to the user guide. GFP–CLU plasmid was generated in the lab^8^. The siRNAs used in the study were: human CLU: 5′- GCAGCAGAGUCUUCAUCAU -3′ (Dharmacon); scramble: 5′- CAGCGCUGACAACAGUUUCAU -3′ (Dharmacon); and ATG3: 5′- GGAAUCAAAGUUUAAGGAAACAGGU -3′ (Invitrogen Life Technologies). CLU antisense oligonucleotide (ASO) OGX-011 was kindly provided by OncoGenex Pharmaceuticals (Vancouver, BC). The sequence of OGX-011 corresponds to the human CLU translation initiation site in exon ll (5′- CAGCAGCAGAGTCTTCATCAT -3′). A scrambled (ScrB, 5′- CAGCGCTGACAACAGTTTCAT -3′) control oligonucleotide was generously provided by ISIS Pharmaceuticals (Carlsbad, CA).

### Western blot analysis

Whole protein lysates were collected using 1 × sample buffer (50 mM Tris-HCl, pH6.8, 2% SDS, 10% glycerol, 0.1 M DTT and 0.02% bromophenol blue) and then separated on 15% (for LC3) or 10% (for all other proteins) polyacrylamide gels. Proteins were transferred to nitrocellulose membranes (Bio-Rad Laboratories, Mississauga, ON) and incubated with 5% skimmed milk to block unspecific signals. Membranes were then incubated with relevant primary antibodies and secondary antibodies as described in ‘Reagents and antibodies’ section, and enhanced chemiluminescence (ECL) were carried out using the reagents from Thermo Scientific (Rockford, IL). For any experiment, when cell death was observed, all floating cells and attaching cells were collected together for lysates preparation. Molecular weight markers are included in all the blots. Full images for key blots were shown in Supplementary Fig. 6.

### Immunoprecipitation

Cells were lysed in NP40 lysis buffer containing protease-inhibitor cocktail (Roche, Indianapolis, IN) and 800 μg proteins were pre-cleared with protein-A/G sepharose (Invitrogen) for 1 h at 4 °C and immunoprecipitated with 4 μg of antibody or immunoglobulin G (IgG) as a negative control for overnight at 4 °C. The immune complexes were recovered with protein-A/G sepharose for 2 h and then washed with NP40 lysis buffer at least three times. The precipitates were boiled with sample buffer for 5 min and proceeded for western blot.

### Immunofluorescence staining and confocal microscopy

Cells grown on glass coverslips were fixed with 3.5% paraformaldehyde and then permeabilized with 0.5% NP40. After blocking with 3% skimmed milk, cells were incubated with primary antibody diluted in 3% milk for 1 h at 37 °C followed by 1 h incubation of the secondary antibody in dark. Coverslips were then mounted on slides with DAPI-containing mounting medium (VECTOR, Burlingame, CA) and stored at +4 °C in dark until being examined with a microscope. Fluorescence images were obtained using a Zeiss LSM 780 confocal microscope (Carl Zeiss, Thornwood, NY). Images were collected using a × 63 1.40 oil Plan-Apochromat DIC M27 Zeiss objective with the confocal pinhole set to 1 Airy unit. For presentation purposes, images were exported as TIFF files. For LC3 puncta, 100 to 200 cells were analysed for each sample from 3 independent experiments. Cells displaying more than 15 bright fluorescent LC3 puncta were counted as positive.

### Transmission electron microscopy

Mice heart tissues were harvested and immediately put into the fixation solution (2.5% glutaraldehyde in 0.1 M sodium cacodylate, pH 7.4). Tissues were cut into 1 mm^3^ tissue blocks under the fixation solution and fixed for another 30 min. After fixation, tissues were maintained in the sodium cacodylate solution until they were processed. Tissues were then post-fixed in 1% osmium tetroxide, dehydrated and embedded in epoxy resin. Ultrathin (90 nm) sections obtained from a Leica UCT ultramicrotome were mounted on formvar carbon-coated cupper grids (Agar Scientific) and stained with uranyl acetate and lead citrate for conventional TEM. All TEM specimens were observed and photographed with a Hitachi H7600 Transmission Electron Microscope. Quantification of autophagic vacuoles was carried out systematically under × 40,000 (refs 58, 59), and images were taken under × 80,000.

### Gel filtration analysis

After treatments, PC3 cells were collected and homogenized in homogenization buffer (40 mM Tris-HCl pH 7.5, 150 mM NaCl and a protease inhibitor mixture from Roche Applied Science) by repeatedly passing (15 times) through a 1 ml syringe with a 23-gauge needle. The homogenate was centrifuged at 13,000*g* for 30 min. The resulting supernatants (2.0 mg protein in 500 μl) were then applied to a Superdex 200 10/300 GL gel filtration column (GE Healthcare) and eluted at a flow rate of 0.5 ml min^−1^ with 40 mM Tris-HCl, pH 7.5 and 150 mM NaCl. Fractions of 500 μl were collected and 20 μl from each fraction was analysed by western blot.

### Site-directed mutagenesis

QuikChange II XL site-directed Mutagenesis Kit (Stratagene, La Jolla, CA, USA) was used to generate CLU mutants according to the instruction manual. Primers used are listed in Supplementary Table S1. The presence of the desired mutations was confirmed by DNA sequencing.

### Flow cytometry analysis

After treatments, cells were collected in phosphate-buffered saline (PBS) and fixed with 70% ethanol for 20 min and then stained with 50 μg ml^−1^ Propidium iodide (PI) in the presence of 0.1 mg ml^−1^ RNase A and 0.05% Triton X-100 for 30 min at 37 °C. Cells were then washed with PBS and applied for flow cytometry analysis (FACSCanto II flow cytometer). The Pre-G1 population was quantified with BD FACSDiva software (version 5.0.3.).

### Cell viability assay

Cell viability assays were performed as described before^55^. Briefly, in the 12-well plates, cells were fixed with 1% glutaradehyde (Sigma, St. Louis, MO) and stained for 5 min with 0.5% crystal violet (Sigma). Plates were washed and air dried, and the dye was eluted with Sorenson’s solution (30 mmol l^−1^ sodium citrate, 0.02 mol l^−1^ HCl and 50% ethanol). Absorbance was determined with a microculture plate reader (Becton Dickinson Labware) at 560 nm, and normalized to values obtained from the vehicle-treated cells to determine percentage change in cell density. Each assay was performed in triplicate.

For clonogenic assay, LNCaP cells (3 × 10^5^) overexpressing CLU or vector alone were seeded in 6-well plates and transfected with siAtg3 or siScramble (siScr). After 2 days of transfection, cells were re-plated in 60 mm tissue culture dishes and then treated with 1 μM of MG132 for 10 days. Culture medium was changed every 5 days. Colonies were visualized by crystal violet staining and relative colony formation was measured at absorbance 560 nm after resolving colonies in Sorenson’s solution, as described above. Three independent experiments have been carried out.

### Assessment of GFP–LC3 puncta *in vivo*

GFP–LC3 mice were kindly provided by Dr. Mizushima (Tokyo Medical and Dental University, Japan)^30^. CLU knockout (*CLU*^*−/−*^) mice in B6 background (males, 8–10 weeks old) were obtained from a breeding colony at the Vancouver Prostate Centre (Vancouver, BC)^60^. GFP–LC3 and *CLU*^*−/−*^ mice were crossed and genotyped using: LC3 primer 1 (5′- ATAACTTGCTGGCCTTTCCACT )-3′, Primer 2 (5′- CGGGCCATTTACCGTAAGTTAT -3′) and Primer 3 (5′- CAGCTCATTGCTGTTCCTCAA -3′); CLU primer 1 (5′- ACGATGTGGAAGGATGTGGAAGATGAACATG -3′), Primer 2 (5′- TGGTGATGGGGCTCTAGTCACCTCCCACTTC -3′) and Primer 3 (5′- CTGCTAAAGCGCATGCTCCAGACTGCCTTG -3′). After treatment, mice heart tissues were collected and fixed with 4% paraformaldehyde dissolved in 0.1 M Na-phosphate buffer (pH 7.4) for 4 h, incubated overnight with 15% sucrose/PBS, and 30% sucrose/PBS for 4 h. Tissues were then embedded in OCT compound (Tissue-Tek) and stored at −80 °C. Sections (5–7 μm) were prepared with a cryostat and air dried at room temperature for 30 min. Images were captured using confocal microscope. Tissues were kept in the dark for most of the steps. All animal procedures were performed according to the guidelines of the Canadian Council on Animal Care and with appropriate institutional certification.

### Assessment of autophagy *in vivo*

Wild-type (wt) C57BL/6j (B6) mice (males, 8 to 10 weeks old) were purchased from the Jackson Laboratory (Bar Harbour, ME), and *CLU*^*−/−*^ mice were from the Vancouver Prostate Centre (Vancouver, BC)^60^. Mice were randomly selected for treatments of CQ+PBS, rapamycin+PBS or rapamycin+CQ, respectively. Rapamycin (10 mg kg^−1^) and CQ (10 mg kg^−1^) were injected intraperitoneally and heart tissues were collected 6 h later for evaluation by western blot analyses for LC3 and TEM analysis for autophagic vacuoles. All the animal procedures were performed according to the guidelines of the Canadian Council on Animal Care with appropriate institutional certification.

### Assessment of *in vivo* tumor growth

For *in vivo* xenograft studies, PC3 cells were inoculated subcutaneously in the flank of 6- to 8-week-old male athymic nude mice (Harlan Sprague Dawley, Inc.) via a 27-gauge needle under isoflurane anaesthesia. When PC3 tumours reached 100 mm^3^, mice were randomly selected for treatments of ScrB+control diluent, ScrB+AZD5363 or OGX-011+AZD5363. AZD5363 (100 mg kg^−1^) was given orally twice a day for 5 days per week, and OGX-011 or ScrB (15 mg kg^−1^) were injected intraperitoneally once daily for 7 days and three times per week thereafter. Tumour volume measurements were performed twice weekly and calculated by the formula length × width × depth × 0.5236. Data points were expressed as average tumour volume±s.e.m. All the animal procedures were performed according to the guidelines of the Canadian Council on Animal Care and with appropriate institutional certification.

### Assessment of *in vivo* tumor pharmacodynamic analysis

PC3 cells were inoculated as described above. When PC3 tumours reached 100 mm^3^, mice were randomly selected for treatments of ScrB+control diluent, ScrB+AZD5363 or OGX-011+AZD5363. AZD5363 (100 mg kg^−1^) was given orally twice a day for 7 days per week, and OGX-011 or ScrB (15 mg kg^−1^) injected intraperitoneally once daily for 7 days. After the treatment, mice were killed and tumours were harvested for the evaluation of protein expression by western blot analyses. All the animal procedures were performed according to the guidelines of the Canadian Council on Animal Care and with appropriate institutional certification.

### Statistical analysis

All the data were analysed by Student’s two-tailed *t*-test. Levels of statistical significance were set at *P*<0.05 or 0.01 as indicated.

## Author contributions

F.Z., M.K. and M.E.G. designed the study. F.Z., M.K., E.B. and S.M. performed the experiments. L.F. assessed animal tissue histology. C.D. involved with GFP–LC3 mice study. P.S. and A.Z. supervised specific experiments and revised the manuscript. F.Z. and M.E.G. wrote the manuscript.

## Additional information

**How to cite this article:** Zhang F. *et al*. Clusterin facilitates stress-induced lipidation of LC3 and autophagosome biogenesis to enhance cancer cell survival. *Nat. Commun.* 5:5775 doi: 10.1038/ncomms6775 (2014).

## Supplementary Material

Supplementary Information Supplementary Figures 1-6 and Supplementary Tables 1, Supplementary Methods

Supplementary Movie 1 PC3 cells growing on glass-base culture dish were co-transfected with GFP-CLU and RFP-LC3 plasmids. One day after transfection, cells were starved with HBSS/HEPES for 2 hours and then live images were taken every 5 seconds with LSM780 microscope under 63X oil objective at 4.8X digital zoom.

## References

[b1] Garrido C. . Heat shock proteins 27 and 70: anti-apoptotic proteins with tumorigenic properties. Cell Cycle 5, 2592–2601 (2006). 17106261 10.4161/cc.5.22.3448

[b2] Kopito R. R. Aggresomes, inclusion bodies and protein aggregation. Trends Cell Biol. 10, 524–530 (2000). 11121744 10.1016/s0962-8924(00)01852-3

[b3] Ding W. X. . Linking of autophagy to ubiquitin-proteasome system is important for the regulation of endoplasmic reticulum stress and cell viability. Am. J. Pathol. 171, 513–524 (2007). 17620365 10.2353/ajpath.2007.070188 PMC1934546

[b4] Zhu K., Dunner K. Jr. & McConkey D. J. Proteasome inhibitors activate autophagy as a cytoprotective response in human prostate cancer cells. Oncogene 29, 451–462 (2010). 19881538 10.1038/onc.2009.343 PMC2809784

[b5] Criswell T. . Delayed activation of insulin-like growth factor-1 receptor/Src/MAPK/Egr-1 signaling regulates clusterin expression, a pro-survival factor. J. Biol. Chem. 280, 14212–14221 (2005). 15689620 10.1074/jbc.M412569200

[b6] Lamoureux F. . Clusterin inhibition using OGX-011 synergistically enhances Hsp90 inhibitor activity by suppressing the heat shock response in castrate-resistant prostate cancer. Cancer Res. 71, 5838–5849 (2011). 21737488 10.1158/0008-5472.CAN-11-0994

[b7] Shiota M. . Clusterin is a critical downstream mediator of stress-induced YB-1 transactivation in prostate cancer. Mol. Cancer Res. 9, 1755–1766 (2011). 21987172 10.1158/1541-7786.MCR-11-0379

[b8] Li N., Zoubeidi A., Beraldi E. & Gleave M. E. GRP78 regulates clusterin stability, retrotranslocation and mitochondrial localization under ER stress in prostate cancer. Oncogene 32, 1933–1942 (2012). 22689054 10.1038/onc.2012.212

[b9] Poon S. . Mildly acidic pH activates the extracellular molecular chaperone clusterin. J. Biol. Chem. 277, 39532–39540 (2002). 12176985 10.1074/jbc.M204855200

[b10] Trougakos I. P. . Intracellular clusterin inhibits mitochondrial apoptosis by suppressing p53-activating stress signals and stabilizing the cytosolic Ku70–Bax protein complex. Clin. Cancer Res. 15, 48–59 (2009). 19118032 10.1158/1078-0432.CCR-08-1805 PMC4483278

[b11] Zhang H. . Clusterin inhibits apoptosis by interacting with activated Bax. Nat. Cell Biol. 7, 909–915 (2005). 16113678 10.1038/ncb1291

[b12] Ammar H. & Closset J. L. Clusterin activates survival through the phosphatidylinositol 3-kinase/Akt pathway. J. Biol. Chem. 283, 12851–12861 (2008). 18321852 10.1074/jbc.M800403200

[b13] Zoubeidi A. . Clusterin facilitates COMMD1 and I-kappaB degradation to enhance NF-kappaB activity in prostate cancer cells. Mol. Cancer Res. 8, 119–130 (2010). 20068069 10.1158/1541-7786.MCR-09-0277 PMC2808437

[b14] Shiota M. . Clusterin mediates TGF-beta-induced epithelial-mesenchymal transition and metastasis via Twist1 in prostate cancer cells. Cancer Res. 72, 5261–5272 (2012). 22896337 10.1158/0008-5472.CAN-12-0254

[b15] Yom C. K., Woo H. Y., Min S. Y., Kang S. Y. & Kim H. S. Clusterin overexpression and relapse-free survival in breast cancer. Anticancer Res. 29, 3909–3912 (2009). 19846927

[b16] July L. V. . Clusterin expression is significantly enhanced in prostate cancer cells following androgen withdrawal therapy. Prostate 50, 179–188 (2002). 11813210 10.1002/pros.10047

[b17] Sowery R. D. . Clusterin knockdown using the antisense oligonucleotide OGX-011 re-sensitizes docetaxel-refractory prostate cancer PC-3 cells to chemotherapy. BJU Int. 102, 389–397 (2008). 18336596 10.1111/j.1464-410X.2008.07618.x

[b18] Zoubeidi A., Chi K. & Gleave M. Targeting the cytoprotective chaperone, clusterin, for treatment of advanced cancer. Clin. Cancer Res. 16, 1088–1093 (2010). 20145158 10.1158/1078-0432.CCR-09-2917 PMC2822877

[b19] Chi K. N. . Randomized phase II study of docetaxel and prednisone with or without OGX-011 in patients with metastatic castration-resistant prostate cancer. J. Clin. Oncol. 28, 4247–4254 (2010). 20733135 10.1200/JCO.2009.26.8771

[b20] Meijer A. J. & Codogno P. Autophagy: regulation and role in disease. Crit. Rev. Clin. Lab. Sci. 46, 210–240 (2009). 19552522 10.1080/10408360903044068

[b21] Mizushima N., Levine B., Cuervo A. M. & Klionsky D. J. Autophagy fights disease through cellular self-digestion. Nature 451, 1069–1075 (2008). 18305538 10.1038/nature06639 PMC2670399

[b22] Gozuacik D. & Kimchi A. Autophagy and cell death. Curr. Top. Dev. Biol. 78, 217–245 (2007). 17338918 10.1016/S0070-2153(06)78006-1

[b23] Bettuzzi S. . Genetic inactivation of ApoJ/clusterin: effects on prostate tumourigenesis and metastatic spread. Oncogene 28, 4344–4352 (2009). 19784068 10.1038/onc.2009.286

[b24] Chayka O. . Clusterin, a haploinsufficient tumor suppressor gene in neuroblastomas. J. Natl Cancer Inst. 101, 663–677 (2009). 19401549 10.1093/jnci/djp063 PMC2720718

[b25] Mizushima N., Yoshimori T. & Levine B. Methods in mammalian autophagy research. Cell 140, 313–326 (2010). 20144757 10.1016/j.cell.2010.01.028 PMC2852113

[b26] He C. & Klionsky D. J. Regulation mechanisms and signaling pathways of autophagy. Annu. Rev. Genet. 43, 67–93 (2009). 19653858 10.1146/annurev-genet-102808-114910 PMC2831538

[b27] Perry C. N. . Novel methods for measuring cardiac autophagy *in vivo*. Methods Enzymol. 453, 325–342 (2009). 19216914 10.1016/S0076-6879(08)04016-0 PMC3658837

[b28] Ni H. M. . Dissecting the dynamic turnover of GFP–LC3 in the autolysosome. Autophagy 7, 188–204 (2011). 21107021 10.4161/auto.7.2.14181 PMC3039769

[b29] Mizushima N. & Kuma A. Autophagosomes in GFP–LC3 Transgenic Mice. Methods Mol. Biol. 445, 119–124 (2008). 18425446 10.1007/978-1-59745-157-4_7

[b30] Kuma A. & Mizushima N. Chromosomal mapping of the GFP–LC3 transgene in GFP–LC3 mice. Autophagy 4, 61–62 (2008). 17786029 10.4161/auto.4846

[b31] Ding W. X. & Yin X. M. Mitophagy: mechanisms, pathophysiological roles, and analysis. Biol. Chem. 393, 547–564 (2012). 22944659 10.1515/hsz-2012-0119 PMC3630798

[b32] Pankiv S. . p62/SQSTM1 binds directly to Atg8/LC3 to facilitate degradation of ubiquitinated protein aggregates by autophagy. J. Biol. Chem. 282, 24131–24145 (2007). 17580304 10.1074/jbc.M702824200

[b33] Mizushima N., Ohsumi Y. & Yoshimori T. Autophagosome formation in mammalian cells. Cell. Struct. Funct. 27, 421–429 (2002). 12576635 10.1247/csf.27.421

[b34] Yamada Y. . The crystal structure of Atg3, an autophagy-related ubiquitin carrier protein (E2) enzyme that mediates Atg8 lipidation. J. Biol. Chem. 282, 8036–8043 (2007). 17227760 10.1074/jbc.M611473200

[b35] Johansen T. & Lamark T. Selective autophagy mediated by autophagic adapter proteins. Autophagy 7, 279–296 (2011). 21189453 10.4161/auto.7.3.14487 PMC3060413

[b36] Behrends C., Sowa M. E., Gygi S. P. & Harper J. W. Network organization of the human autophagy system. Nature 466, 68–76 (2010). 20562859 10.1038/nature09204 PMC2901998

[b37] Kraft C., Peter M. & Hofmann K. Selective autophagy: ubiquitin-mediated recognition and beyond. Nat. Cell Biol. 12, 836–841 (2010). 20811356 10.1038/ncb0910-836

[b38] Chen N. & Debnath J. Autophagy and tumorigenesis. FEBS Lett. 584, 1427–1435 (2010). 20035753 10.1016/j.febslet.2009.12.034 PMC2843775

[b39] Ryter S. W., Cloonan S. M. & Choi A. M. Autophagy: a critical regulator of cellular metabolism and homeostasis. Mol. Cells 36, 7–16 (2013). 23708729 10.1007/s10059-013-0140-8 PMC3887921

[b40] Nakatogawa H., Suzuki K., Kamada Y. & Ohsumi Y. Dynamics and diversity in autophagy mechanisms: lessons from yeast. Nat. Rev. Mol. Cell Biol. 10, 458–467 (2009). 19491929 10.1038/nrm2708

[b41] Roy S. & Debnath J. Autophagy and tumorigenesis. Semin. Immunopathol. 32, 383–396 (2010). 20589500 10.1007/s00281-010-0213-0 PMC2999728

[b42] Liang X. H. . Induction of autophagy and inhibition of tumorigenesis by beclin 1. Nature 402, 672–676 (1999). 10604474 10.1038/45257

[b43] Hu Y. L. . Hypoxia-induced autophagy promotes tumor cell survival and adaptation to antiangiogenic treatment in glioblastoma. Cancer Res. 72, 1773–1783 (2012). 22447568 10.1158/0008-5472.CAN-11-3831 PMC3319869

[b44] Shi Z. . A systems biology analysis of autophagy in cancer therapy. Cancer Lett. 337, 149–160 (2013). 23791881 10.1016/j.canlet.2013.06.004

[b45] Boutin B. . Androgen deprivation and androgen receptor competition by bicalutamide induce autophagy of hormone-resistant prostate cancer cells and confer resistance to apoptosis. Prostate 73, 1090–1102 (2013). 23532738 10.1002/pros.22658

[b46] Lamoureux F. . Blocked autophagy using lysosomotropic agents sensitizes resistant prostate tumor cells to the novel Akt inhibitor AZD5363. Clin. Cancer Res. 19, 833–844 (2013). 23258740 10.1158/1078-0432.CCR-12-3114

[b47] Kimura T., Takabatake Y., Takahashi A. & Isaka Y. Chloroquine in cancer therapy: a double-edged sword of autophagy. Cancer Res. 73, 3–7 (2013). 23288916 10.1158/0008-5472.CAN-12-2464

[b48] Solomon V. R. & Lee H. Chloroquine and its analogs: a new promise of an old drug for effective and safe cancer therapies. Eur. J. Pharmacol. 625, 220–233 (2009). 19836374 10.1016/j.ejphar.2009.06.063

[b49] Wyatt A. R. . Clusterin facilitates *in vivo* clearance of extracellular misfolded proteins. Cell. Mol. Life. Sci. 68, 3919–3931 (2011). 21505792 10.1007/s00018-011-0684-8 PMC11115182

[b50] Jones S. E. & Jomary C. Clusterin. Int. J. Biochem. Cell Biol. 34, 427–431 (2002). 11906815 10.1016/s1357-2725(01)00155-8

[b51] Carver J. A., Rekas A., Thorn D. C. & Wilson M. R. Small heat-shock proteins and clusterin: intra- and extracellular molecular chaperones with a common mechanism of action and function? IUBMB Life 55, 661–668 (2003). 14769002 10.1080/15216540310001640498

[b52] Rozenknop A. . Characterization of the interaction of GABARAPL-1 with the LIR motif of NBR1. J. Mol. Biol. 410, 477–487 (2011). 21620860 10.1016/j.jmb.2011.05.003

[b53] Nezis I. P. & Stenmark H. p62 at the interface of autophagy, oxidative stress signaling, and cancer. Antioxid. Redox Signal 17, 786–793 (2012). 22074114 10.1089/ars.2011.4394

[b54] Su H. & Wang X. p62 Stages an interplay between the ubiquitin-proteasome system and autophagy in the heart of defense against proteotoxic stress. Trends Cardiovasc. Med. 21, 224–228 (2011). 22902070 10.1016/j.tcm.2012.05.015 PMC3424486

[b55] Matsumoto H. . Cotargeting androgen receptor and clusterin delays castrate-resistant prostate cancer progression by inhibiting adaptive stress response and AR stability. Cancer Res. 73, 5206–5217 (2013). 23786771 10.1158/0008-5472.CAN-13-0359

[b56] Ammar H. & Closset J. L. Clusterin activates survival through the phosphatidylinositol 3-kinase/Akt pathway. J. Biol. Chem. 283, 12851–12861 (2008). 18321852 10.1074/jbc.M800403200

[b57] Miyake H., Nelson C., Rennie P. S. & Gleave M. E. Testosterone-repressed prostate message-2 is an antiapoptotic gene involved in progression to androgen independence in prostate cancer. Cancer Res. 60, 170–176 (2000). 10646870

[b58] Yla-Anttila P., Vihinen H., Jokitalo E. & Eskelinen E. L. Monitoring autophagy by electron microscopy in Mammalian cells. Methods Enzymol. 452, 143–164 (2009). 19200881 10.1016/S0076-6879(08)03610-0

[b59] Eskelinen E. L. Fine structure of the autophagosome. Methods Mol. Biol. 445, 11–28 (2008). 18425441 10.1007/978-1-59745-157-4_2

[b60] Li S. . Reduction of cold ischemia-reperfusion injury by graft-expressing clusterin in heart transplantation. J Heart Lung Transplant 30, 819–826 (2011). 21515078 10.1016/j.healun.2011.03.007

