# Supplementary material for: Clusterin facilitates stress-induced lipidation of LC3 and autophagosome biogenesis to enhance cancer cell survival
Source: Nat Commun. 2014 Dec 12;5:5775. doi: 10.1038/ncomms6775 (PMC4275590; doi:10.1038/ncomms6775)
Supplement: Supplementary Information — Supplementary Figures 1-6 and Supplementary Tables 1, Supplementary Methods [file ncomms6775-s1.pdf]

Supplementary Fig. 1

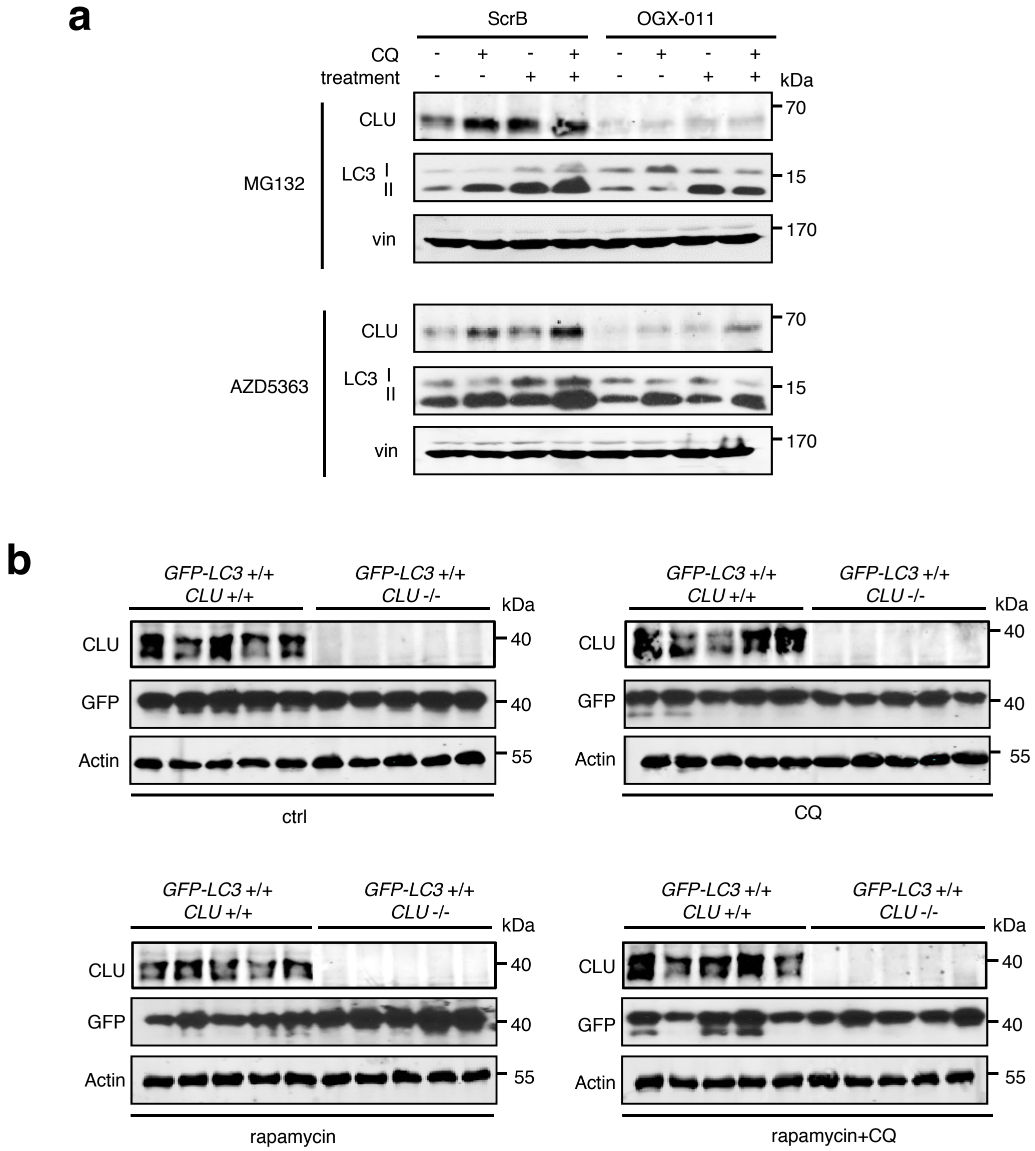

Supplemental Fig. 1. Loss of CLU reduces autophagy activity. (**a**) PC3 cells were treated with CLU antisense OGX-011 or scrambled antisense (ScrB) followed with 10 µM MG132 or 10µM AZD5363 treatments for 6 hrs with or without CQ. Whole protein lysates were collected for western blot against CLU and LC3. (**b**) Heart tissues from mice treated as in figure 2f were collected to prepare whole protein lysates. CLU and GFP protein levels were analyzed using western blots. The lower bands from GFP blots represent GFP-LC3**II** protein.

Supplementary Fig. 2

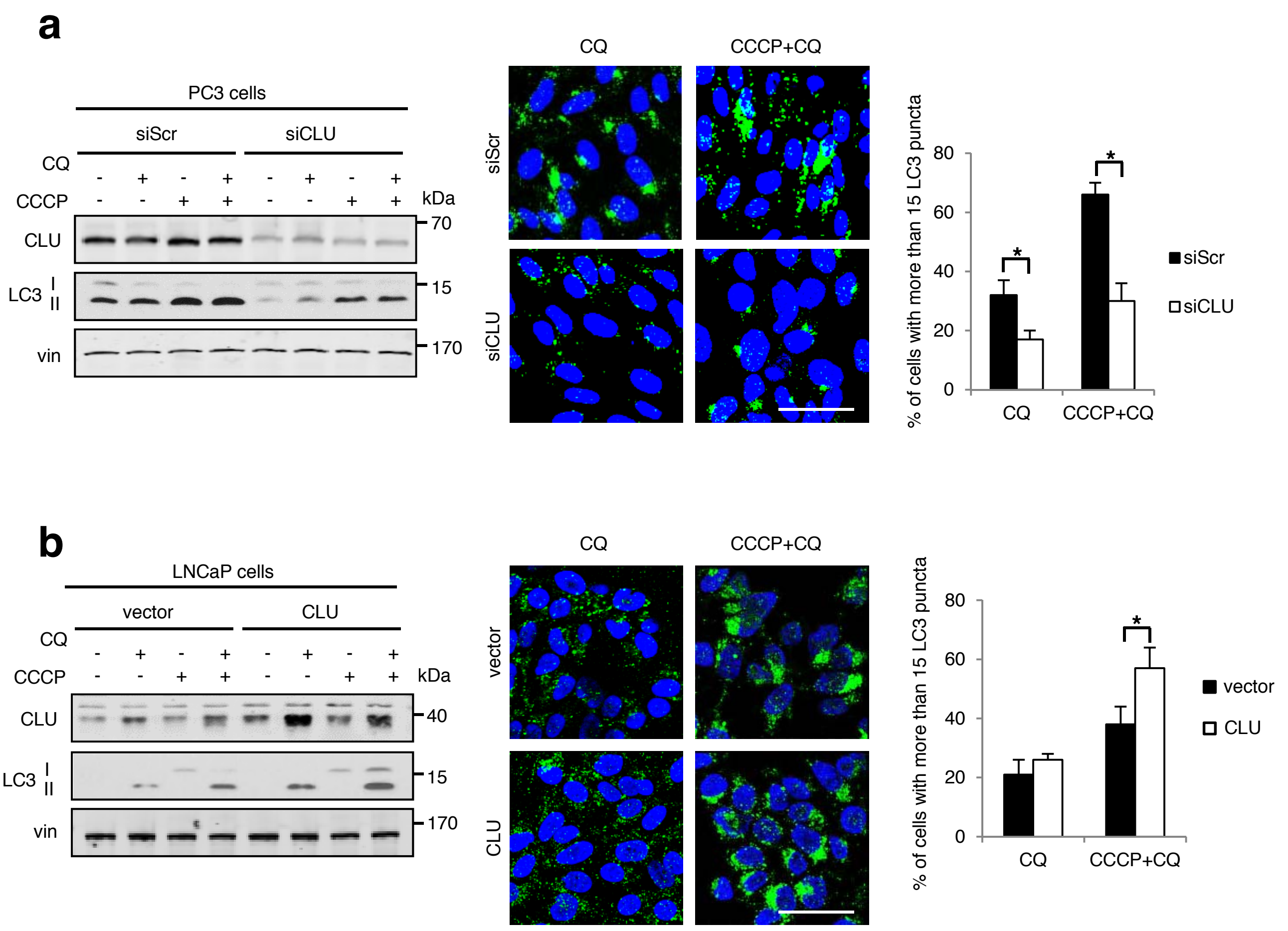

Supplemental Fig. 2. CLU modulates mitophagy. (**a**) PC3 cells transfected with siCLU or ctrl siScr were treated with mitophagy inducer carbonyl cyanide m-chlorophenylhydrazone (CCCP) with or without CQ for 6 hrs. Autophagy activity was analyzed using LC3 western blot and puncta assay. Scale bar: 50 µm. (**b**) LNCaP cells overexpressing CLU or vector alone were treated with CCCP for 24 hours with or without CQ . Autophagy activity was measured as (**a**). Scale bar: 50 µm. For all panels, *p<0.05 (Student's two-tailed *t*-test of three experiments). Error bars: s.e.m of at least three experiments.

Supplementary Fig. 3

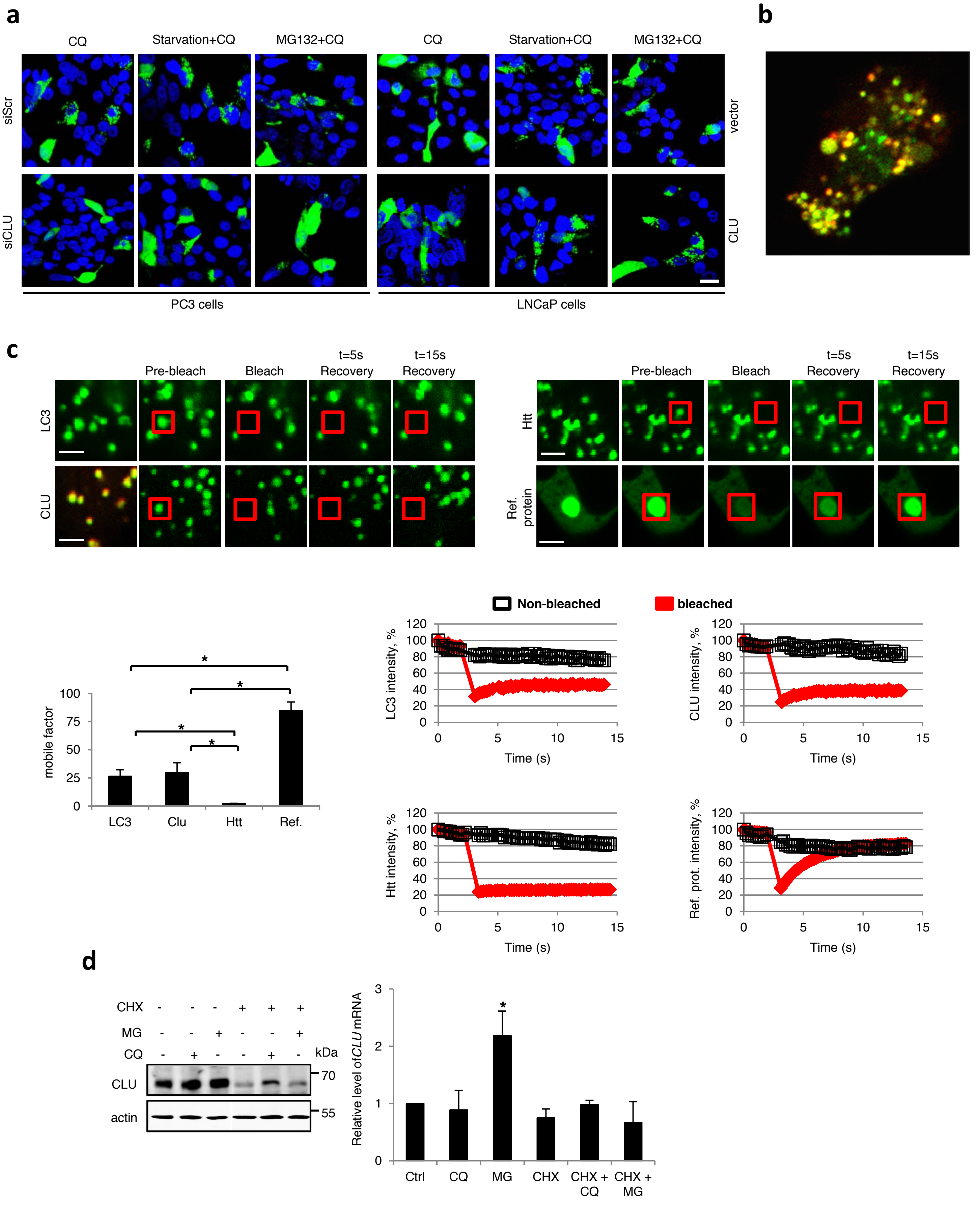

Supplementary Fig. 3

Supplemental Fig. 3. CLU co-localizes and moves together with LC3II. (**a**) PC3 cells (left panel) treated with siCLU or siScr were transfected with GFP-LC3 plasmid followed by starvation +CQ or MG132 +CQ for 6 hrs. LNCaP cells overexpressing CLU or vector alone (right panel) were treated for 24 hrs. GFP-LC3 puncta were analyzed under confocal microscopy. Scale bar: 50 µm. (**b**) PC3 cells were transfected with GFP-CLU and RFP-LC3 followed by starvation for 2 hrs. Cells were then examined under microscope and the live images were taken every 5 seconds for 3 minutes. (**c**) PC3 cells transfected with GFP-tagged proteins were starved for 2 hrs and then applied for FRAP assay. The fluorescence of GFP proteins was shown, and the mobile factors were calculated with the software ZEN2010 from Carl Zeiss from at least 5 cells for each samples. The red box represented the bleached region. GFP-tagged mutated huntingtin (Htt) and a reference GFP-tagged protein were included as control. *$p<0.001$ (Student's two-tailed *t*-test of three experiments). Error bars: s.e.m of at least three experiments. Scale bar: 5 µm. (**d**) PC3 cells were treated with 10 µM MG132 (MG) or 10 µM CQ with or without cycloheximide (CHX) for 6 hrs. Protein lysates were collected for analysis on CLU. In the right panel, mRNA was prepared for the quantitative-PCR assay on *CLU*. *$p<0.05$ (Student's two-tailed *t*-test of three experiments). Error bars: s.e.m of at least three experiments.

Supplementary Fig. 4

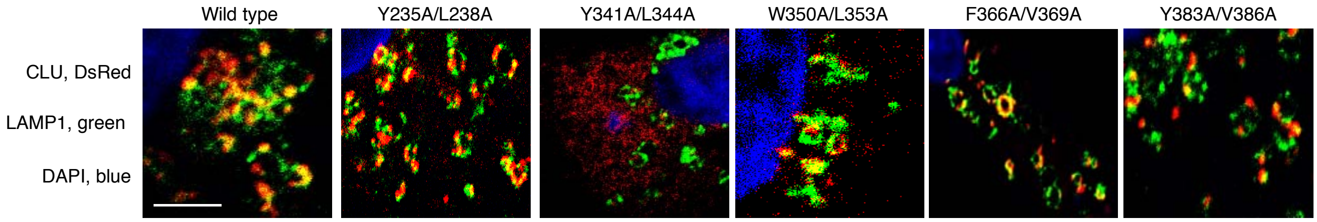

Supplemental Fig. 4. CLU LIR mutant does not bind to LAMP1. PC3 cells were transfected with DsRed labeled-wild type or mutant CLU followed by 4 hrs treatment with 10 µM MG132 with CQ. LAMP1 immunofluorescence staining (green) was performed. Images were scanned using LSM780 confocal microscope. Scale bar: 5 µm.

Supplementary Fig. 5

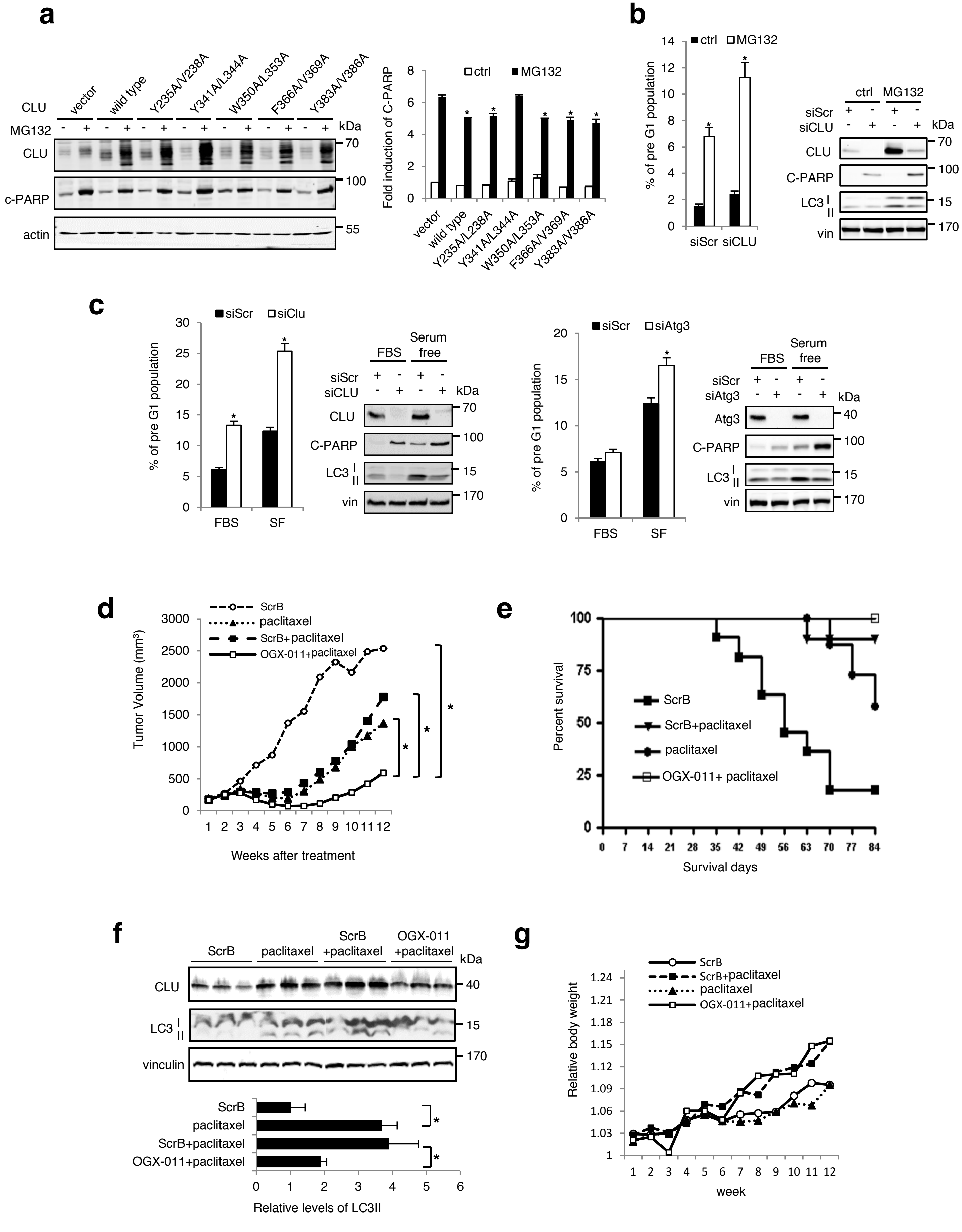

Supplementary Fig. 5

Supplemental Fig. 5. CLU mediates cytoprotection in an autophagy-dependent manner and CLU inhibition sensitizes autophagy-inducing treatments. (**a**) LNCaP cells expressing wild type CLU or mutants were treated with MG132 for 24 hours. Whole protein lysates were collected to check protein levels of CLU and cleaved-PARP (C-PARP). Levels of C-PARP were quantified and fold of induction compared to ctrl were shown in the right panel. *$p<0.05$ (Student's two-tailed *t*-test of three experiments). Error bars: s.e.m of at least three experiments. (**b**) PC3 cells transfected with siCLU or siScr were treated with MG132 for 24 hours. Cell death was investigated using FACS to analyze the pre-G1 population on the left panel. *$p<0.01$ (Student's two-tailed *t*-test of three experiments). Autophagy level and cell death status were examined using western blot against LC3 and cleaved PARP (right panel). (**c**) In the left panel, CLU was knocked down in PC3 cells followed by serum starvation (serum free) treatment. Autophagy activation and cell death were investigated as (**b**). In the right panel, PC3 cells were treated with siAtg3 or siScr followed by serum starvation. Autophagy activation and cell death were investigated as in the left panel. *$p<0.01$ (Student's two-tailed *t*-test of three experiments). (**d**) When PC3 xenografts reached $100mm^3$, mice were treated with ScrB, paclitaxel, ScrB+paclitaxel, or OGX-011+paclitaxel for 12 weeks. Tumor volumes were measured weekly and calculated by length x width x depth x 0.5236. *$p<0.05$ (Student's two-tailed *t*-test). (**e**) Kaplan Meier survival curves of mice treated as in (**d**), n=10. *$p=0.0001$, log-rank test. (**f**) Western blot analysis on CLU and LC3II from tumor protein lysates. LC3II protein levels were quantified after balanced with vinculin. *$p<0.05$ (Student's two-tailed *t*-test). (**g**) Body weights of mice treated in (**d**) were measured weekly and were presented as relative body weight as compared to the first week's measurement. No significant differences cross groups were detected.

Supplementary Fig. 6

Fig. 1a

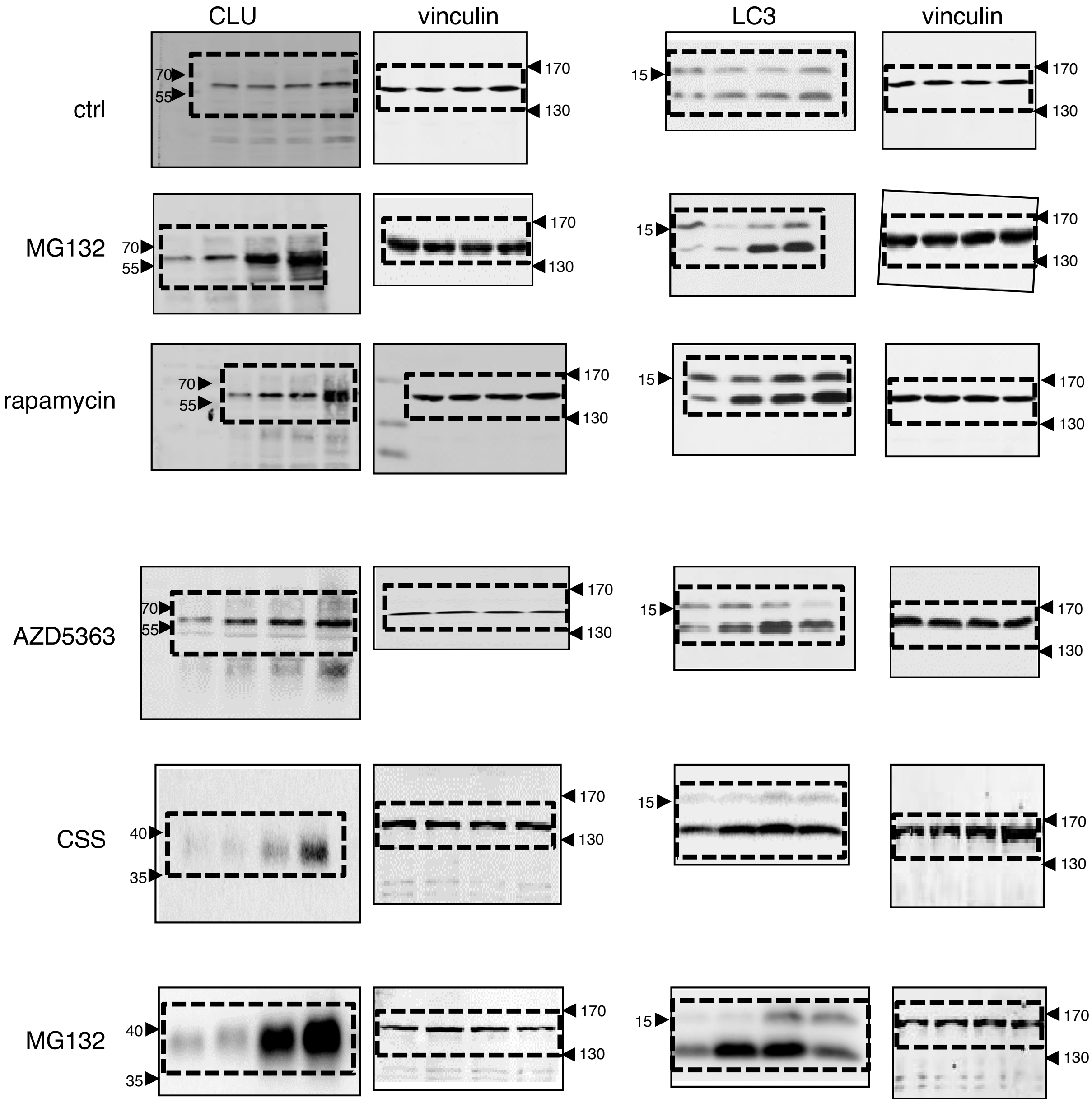

Supplemental Fig. 6. Original immunoblot data. The numbers besides the black arrows show the molecular weight (kDa).

Supplementary Fig. 6

Fig. 2a

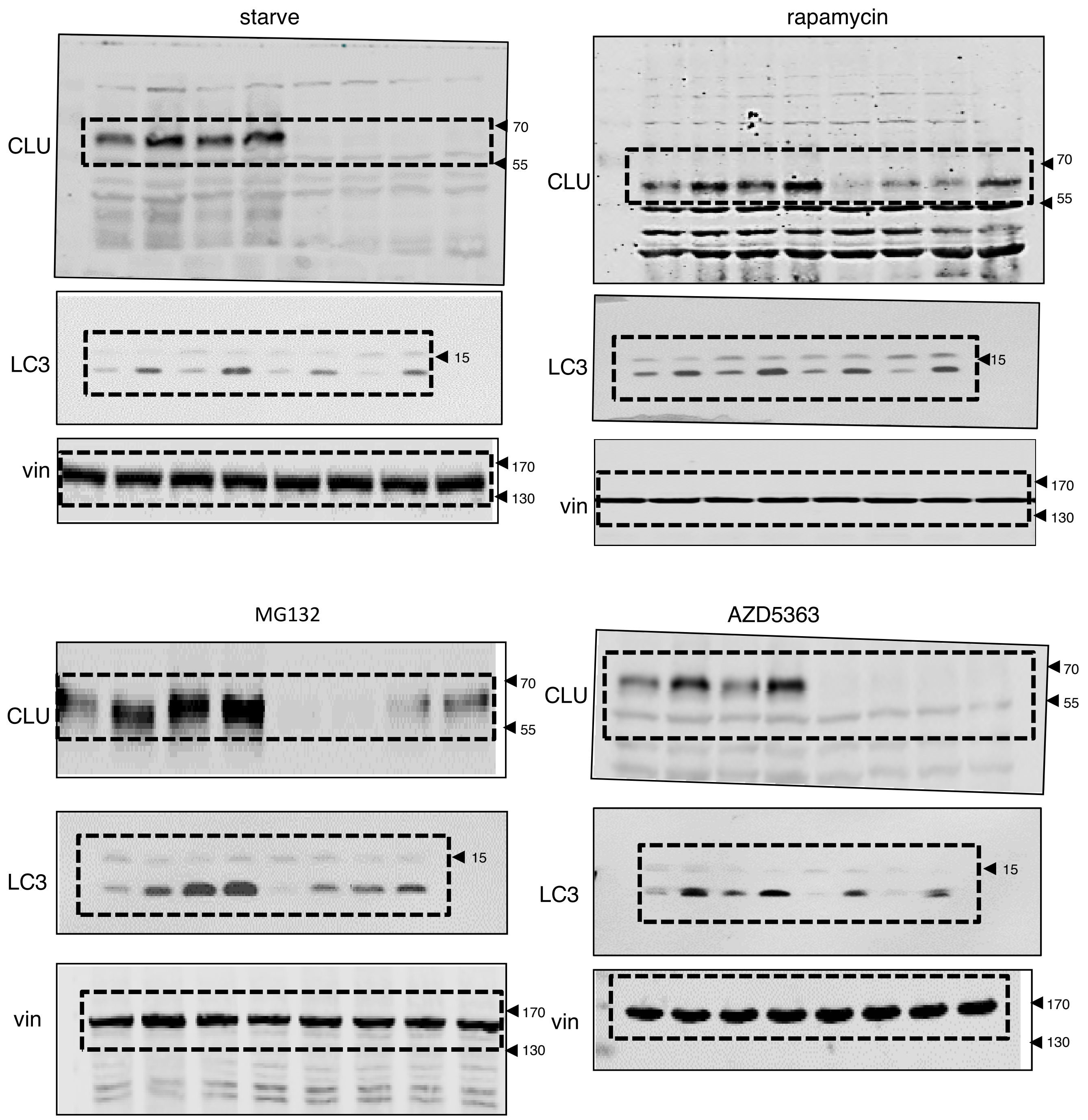

Supplemental Fig. 6. Original immunoblot data. Continued.

Supplementary Fig. 6

Fig. 3a

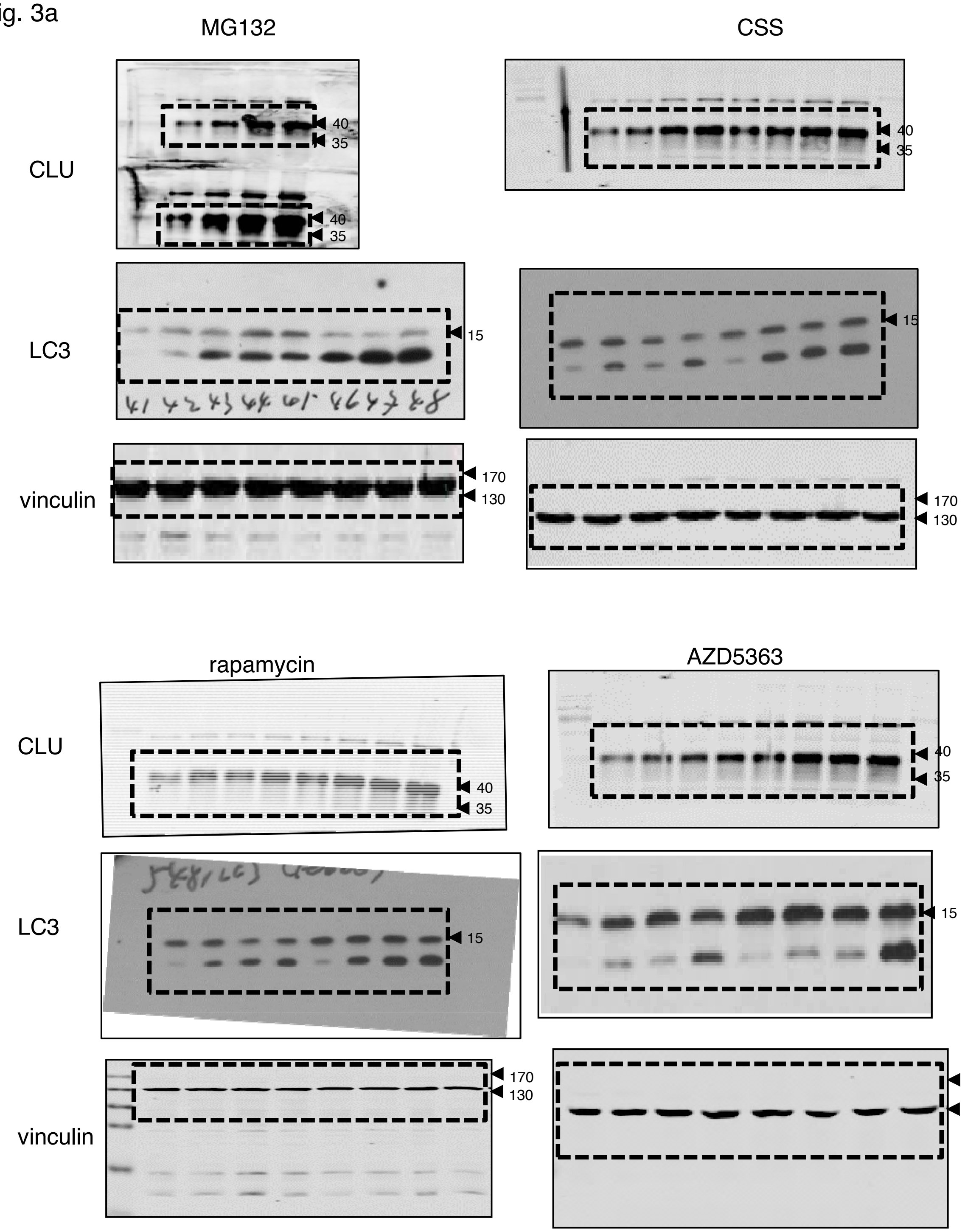

Supplemental Fig. 6. Original immunoblot data. Continued.

Supplementary Fig. 6

Fig. 4e

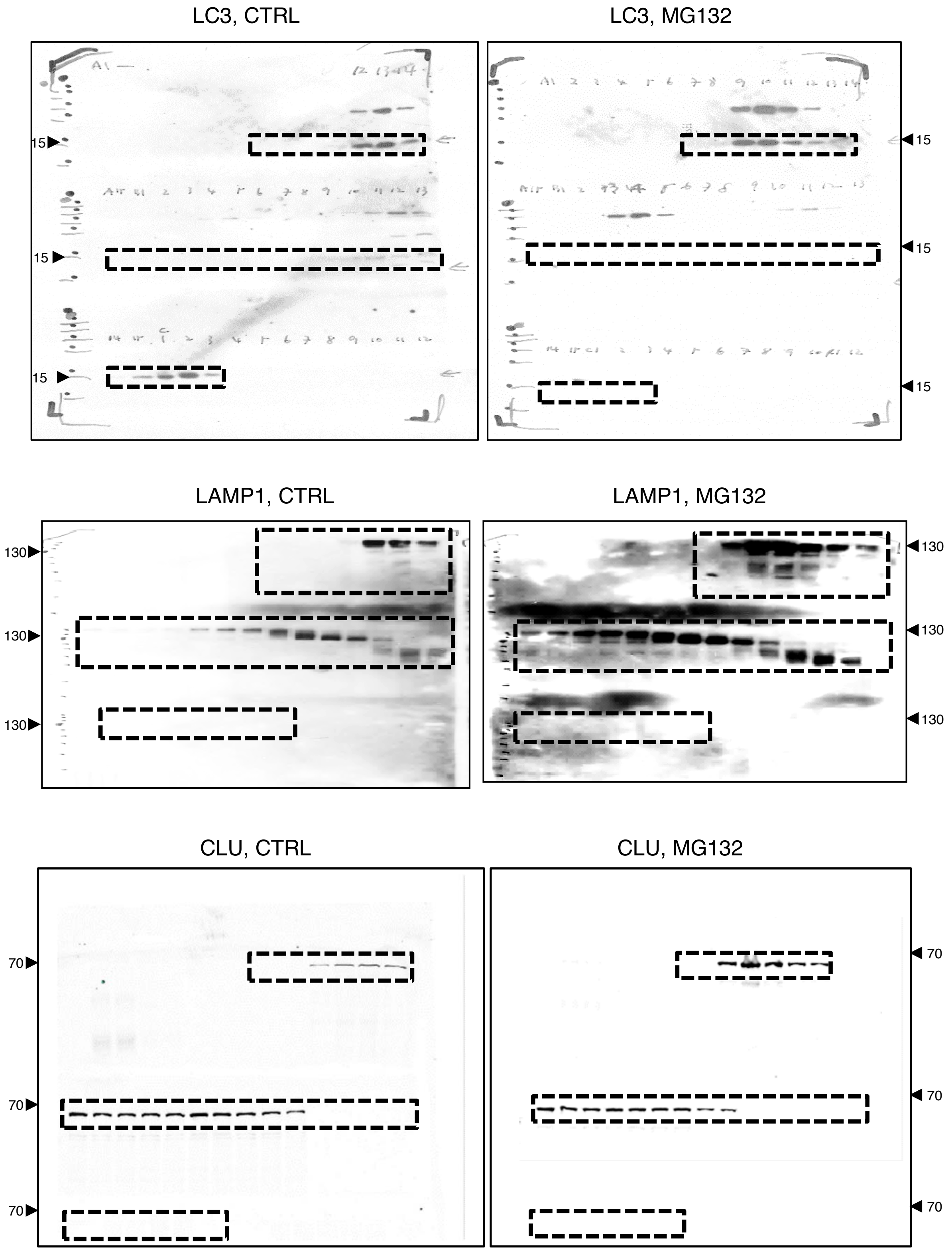

Supplemental Fig. 6. Original immunoblot data. Continued.

Supplementary Fig. 6

Fig. 5b

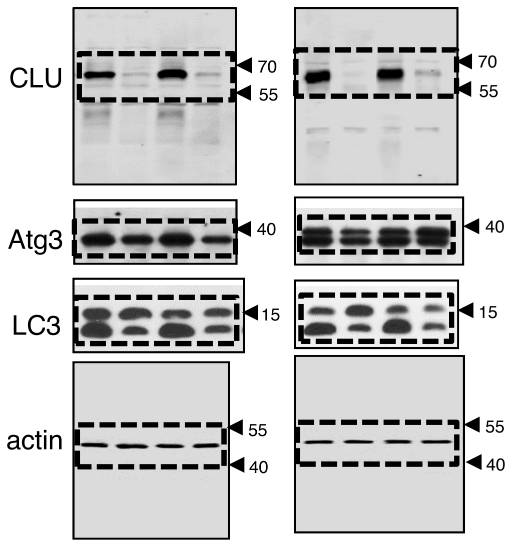

Fig. 5c&d

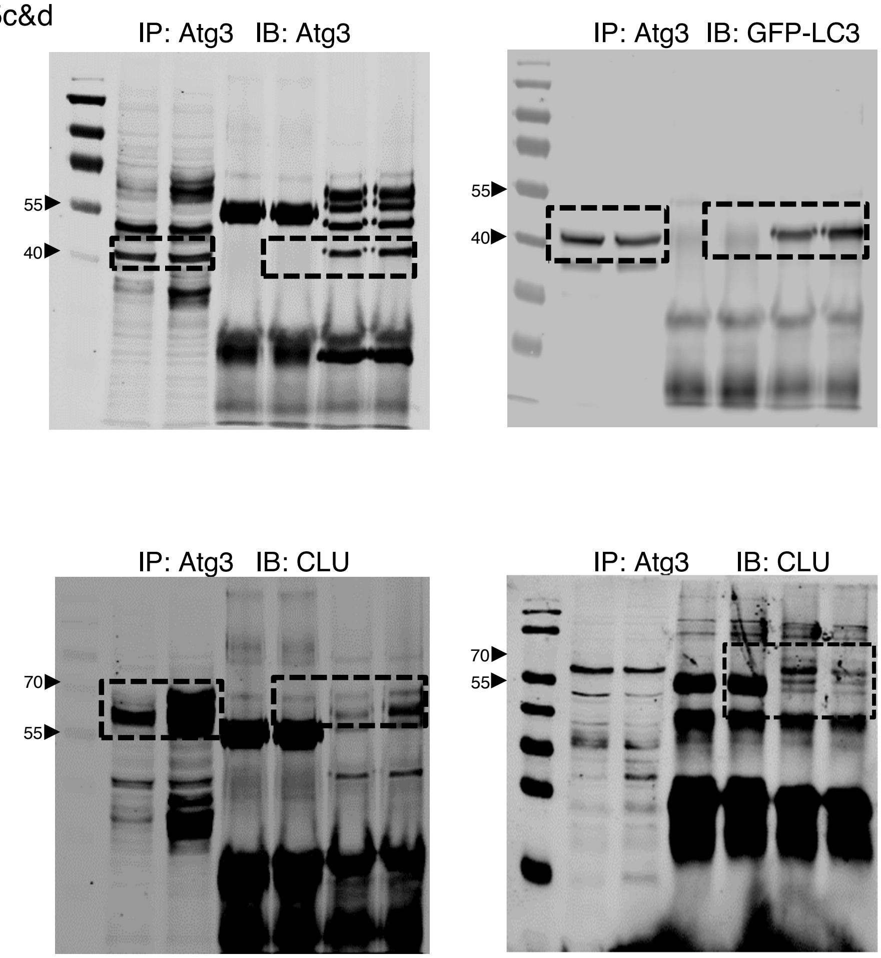

Supplemental Fig. 6. Original immunoblot data. Continued.

Supplementary Fig. 6

Fig. 6c

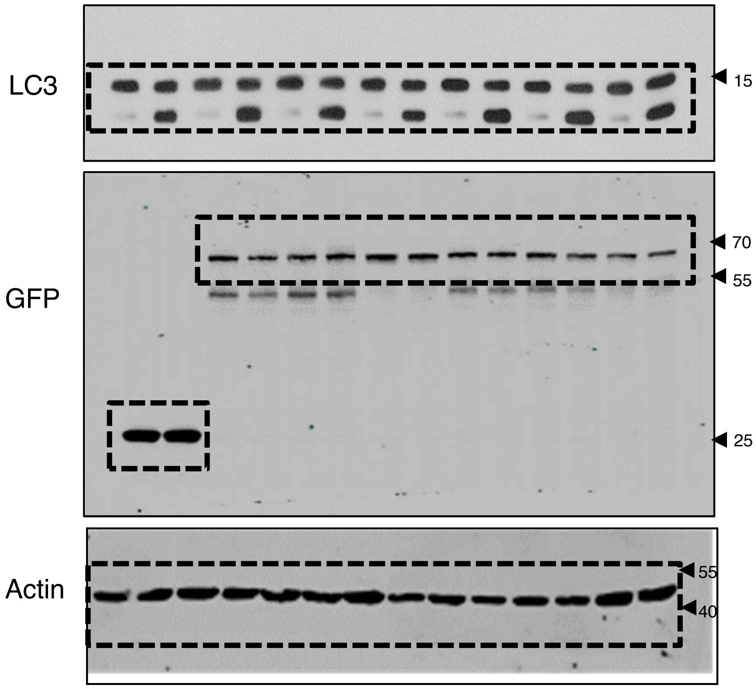

Supplemental Fig. 6. Original immunoblot data. Continued.

Supplementary Table 1. Primers used for subcloning of wild type CLU and mutants

wild type CLU	forward	5'-tttaagcttatgatgaagactctgct-3'
	backward	5'-tggatccttctcctcccggtgctt-3'
Y235A/L238A	forward	5'-gcccttctctccggccgagcccgcgaacttccacgcc-3'
	Backward	5'ggcgtggaagttcgcgggctcggccggagagaagggc3'
Y341A/L344A	forward	5'-gctgagaggttgaccaggaaagccaacgaggcgctaaagtcctaccag-3'
	Backward	5'-ctggtaggactttagcgcctcgttggctttcctggtcaacctctcagc-3'
W350A/L353A	forward	5'-ctgctaaagtcctaccaggcgaagatggccaacacctcctccttgct-3'
	Backward	5'-agcaaggaggaggtgttggccatcttcgcctggtaggactttagcag-3'
F366A/V369A	forward	5'-gctgaacgagcaggctaactgggcgtcccggctggca-3'
	Backward	5'-tgccagccgggacgcccagttagcctgctcgttcagc-3'
Y383A/V386A	forward	5'-cgaagaccagtacgctctgcgggccaccacggtggct-3'
	backward	5'-agccaccgtggtggcccgcagagcgtactggtcttcg-3'

Supplementary methods

Live-cell imaging. GFP-CLU and RFP-LC3 plasmids were co-transfected into PC3 cells cultured on a glass-base culture dish and maintained in phenol-red free DMEM containing 10% fetal calf serum. 24 hours later, cells were starved with HBSS/Hepes for 2 hours and then being imaged with LSM780 microscope under 63X 1.40 oil Plan-Apochromat DIC M27 Zeiss objective at 4.8X digital zoom. Live images were taken every 5 seconds and the movie shown here is a 90 seconds film.

Fluorescence recovery after photobleaching (FRAP). PC3 cells cultured on a glass-base culture dish were transfected with GFP-tagged proteins. Imaging were captured every 0.2 seconds using a 63X 1.40 oil Plan-Apochromat DIC M27 Zeiss objective at 10X digital zoom. After 10 cycles, the red frame region was photobleached with 100% laser power for repetitively scanning for 40 times. After that, images were collected every 0.2 seconds during the recovery phase for a total of 15 seconds. The fluorescence of a non-bleached region was also captured from the same field for reference purpose. The mobile factor was calculated by comparing the fluorescence intensity of the bleached region to the non-bleached region by the software ZEN2010 from Zeiss (Thornwood, NY).

***In vivo* tumor growth and Kaplan Meier Survival Analysis.** PC3 cells (6 million) were inoculated s.c. in the flank of 6- to 8-week-old male athymic nude mice (Harlan Sprague Dawley, Inc.) via a 27-gauge needle under isoflurane anesthesia. When PC3 tumors reached 100 mm^3, mice were randomly selected for treatments of scramble (ScrB), paclitaxel, ScrB+paclitaxel, or OGX-011+paclitaxel. Paclitaxel (0.5mg/kg) was injected i.p. to mice three times a week for every

two weeks; and OGX-011 or ScrB (15 mg/kg) was injected i.p. once daily for 7 days and then three times per week thereafter. Tumor volume measurements were performed weekly and calculated by the formula length x width x depth x 0.5236. Data points were expressed as average tumor volume ± s.e.m. All animal procedures were performed according to the guidelines of the Canadian Council on Animal Care and with appropriate institutional certification. The Kaplan Meier survival curve with log-rank analysis was performed using GraphPad Prism (version 4.00 for Windows, GraphPad Software, San Diego California USA).
